# Molecular Pseudorotation in Phthalocyanines as a Tool for Magnetic Field Control at the Nanoscale

**DOI:** 10.1021/jacs.4c01915

**Published:** 2024-05-14

**Authors:** Raphael Wilhelmer, Matthias Diez, Johannes K. Krondorfer, Andreas W. Hauser

**Affiliations:** Institute of Experimental Physics, Graz University of Technology, Petersgasse 16, A-8010 Graz, Austria

## Abstract

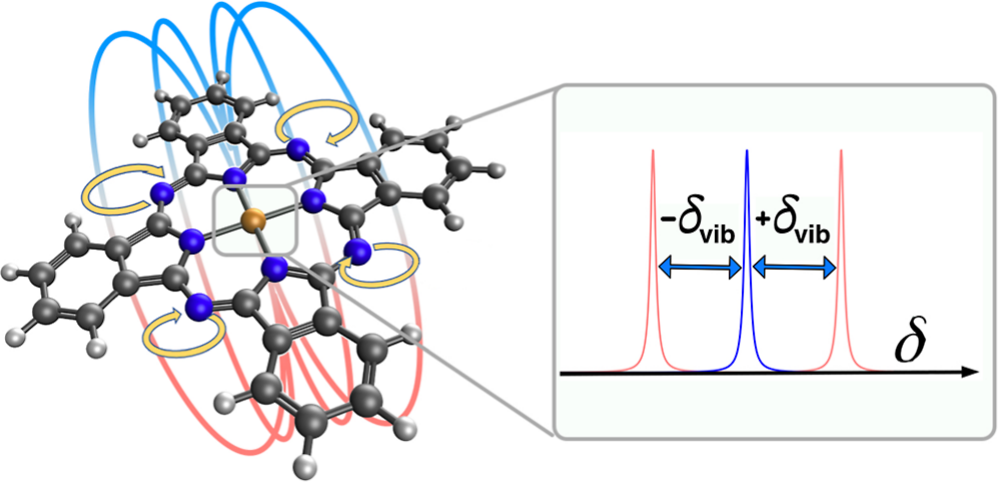

Metal phthalocyanines, a highly versatile class of aromatic, planar, macrocyclic molecules with a chelated central metal ion, are topical objects of ongoing research and particularly interesting due to their magnetic properties. However, while the current focus lies almost exclusively on spin-Zeeman-related effects, the high symmetry of the molecule and its circular shape suggests the exploitation of light-induced excitation of 2-fold degenerate vibrational states in order to generate, switch, and manipulate magnetic fields at the nanoscale. The underlying mechanism is a molecular pseudorotation that can be triggered by infrared pulses and gives rise to a quantized, small, but controllable magnetic dipole moment. We investigate the optical stimulation of vibrationally induced molecular magnetism and estimate changes in the magnetic shielding constants for confirmation by future experiments.

## 1 Introduction

The manipulation of organic molecules by magnetic fields is a highly active field of research with potential applications in molecular circuitry,^1,2^ quantum computing,^3,4^ optoelectronics,^5^ and even the chiral separation of racemic mixtures via enantiospecific induced spin polarization.^6,7^ Typically, paramagnetic states with nonzero spin multiplicity are the starting point for investigations of magnetic field sensitivity. When exposed to an external magnetic field, the degeneracy of the orientational spin quantum number is lifted, and changing the occupation of these magnetic sublevels is known to affect the chemical, optical and electronic properties of certain materials,^8−12^ a concept that has been exploited by nature on numerous occasions in the form of biological sensors using earth’s magnetic field for orientation.^13−15^

Nevertheless, on the road toward new multifunctional organic devices with integrated electronic, optical, and magnetic properties, studies on the diamagnetic properties of these materials have seen a very recent revival, in particular with respect to the phenomenon of ring currents of aromatic molecules.^16,17^ Although the actual understanding of ring currents in chemical systems is still incomplete,^5,18−20^ their consequences in nuclear magnetic resonance (NMR) spectroscopy are well studied.^21,22^ A particularly interesting question is whether additional ring currents, driven by an external magnetic field, can also be used to control molecular features. Recently, a large step in this direction has been made by Kudisch et al., who demonstrated a change in the photophysical properties of π-stacked H_2_–phthalocyanine molecules when exposed to a strong magnetic field.^5^ The latter research is particularly noteworthy as most of the ongoing work on phthalocyanines is dedicated only to spin Zeeman coupling effects in the subclass of metal phthalocyanines. This abundant group of molecules has become one of the most studied organic materials for applications as catalysts, dyes, and coatings due to their optical, magnetic, and electronic properties. It has been studied in the bulk, as thin films, and in solution as well as on single molecules on various substrates.^23−25^

**Figure 1:**
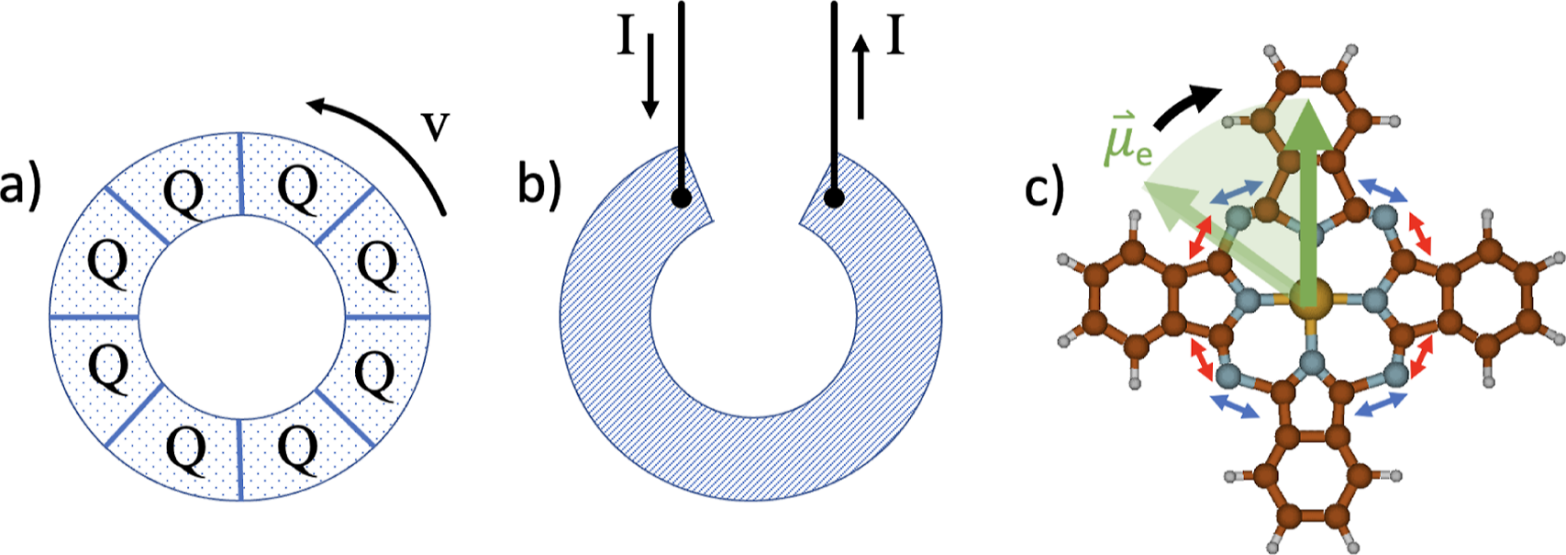
Three physical setups that create a magnetic field perpendicular to the paper plane: (a) Rowland’s experiment of a rotating ring of charges, (b) a current *I* passing through a loop of conductive material, and (c) a metal phthalocyanine, periodically deformed by two degenerate vibrations (red and blue, here mostly affecting outer N positions), giving rise to a rotation of an electric dipole moment (green) in the molecular plane.

In this article, although closely related to the above in terms of its consequences, an entirely different handle on ring currents and magnetism of aromatic molecules is proposed by theory: a “concerted”, two-dimensional molecular vibration, also known as pseudorotation. The latter term is commonly used to describe an intramolecular motion, resulting in a structure that appears to have been produced by a rotation of the entire initial molecule. Within the picture of vibrational eigenstates, motions of this type may be interpreted as simultaneous excitation of energetically degenerate vibrational modes. As is shown below, the linear combination of two degenerate, infrared (IR) active molecular vibrations, excited with a phase delay of π/2, can cause a pseudorotational motion, which gives rise to an electric dipole moment vector that rotates in the molecular plane and may be accompanied by a vibrationally induced intramolecular magnetic field. From an oversimplified but intuitive perspective, such a specific vibrational excitation may turn suitable molecules into a molecular analogue of Rowland’s famous ring experiment,^26^ as is shown in Figure 1. Regarding the magnetic field created by this contraption, its similarity to the field caused by a rotation of isolated charges on a ring, or a current flowing through a single loop of conducting wire, will be investigated in detail. We discuss the suitability of empirical and quantum-mechanical models in an attempt to describe electric dipole moment dynamics, an effective magnetic dipole moment, and the generation of an intramolecular vibrationally induced magnetic field.

While the combination of degenerate vibrational excitations with a specific phase relation has been a commonplace in theoretical molecular spectroscopy for decades,^27−75^ e.g., to capture intramolecular rearrangements such as the name-giving Berry mechanism in highly symmetric molecules,^29^ experimental control and exploitation has come into reach just recently by the advent of ultrashort laser spectroscopy.^30−32^ However, most often, the focus is set on electronic degeneracies in aromatic molecules^33−36^ and hence on spectroscopy in the ultraviolet/vis regime. Very closely related is the work of Juraschek et al.^37−39^ on phonon-mediated dynamical multiferroicity, where a time-dependent polarization is proposed to induce magnetization in bulk materials.

Our article is structured as follows. In the first section, we provide an abbreviated review of the rotational *g*-factor, a dimensionless quantity connecting the angular momentum and magnetic moment, and its related spin-rotation coupling parameters. This is followed by the extension of the existing theories on the vibrational *g*-factor, in particular the semi-classical model of Moss and Perry,^40^ to capture spin–vibration coupling as well, within a unified treatment for rotation and vibration. We investigate the coupling among molecular pseudorotation, an external magnetic field, and the nuclear spins within a given molecule. The underlying principle is the fact that pseudorotations involve the motion of certain atomic nuclei along closed loops and, as charge-carrying particles, they may generate magnetic fields.

In the main part, H_2_Pc and CuPc molecules, two extreme representatives of the metal phthalocyanine complexes, are investigated in greater detail via density functional theory (DFT). The GFN2-*x*TB method, a tight binding ansatz of considerably reduced computational cost, is validated through direct comparison on these two molecules and applied to Mn–Pc, Fe–Pc, Co–Pc, and Ni–Pc in the Supporting Information. Given our interest in the possibility of photoexcitation of magnetic field-generating vibrational modes, we present IR spectra and select the strongest, IR-active planar pseudorotations for further study. Vibrational *g*-factors are then calculated, and changes in the dipole moment as well as the overall electron density distribution are presented as a function of the corresponding concerted vibrational motion. A particularly well-suited type of pseudorotation, typically located at about 1526 cm^–1^ in all phtalocyanines under study, is selected for further investigation, and changes of the magnetic shielding constants in both directions, triggered by the optical excitation, are estimated for future experimental verification of vibrationally induced local magnetic fields.

## 2 Methodology

### 2.1 Magnetic Effects due to Molecular Pseudorotation

Cyclic motions in molecules or solids, where the positively charged nuclei are moving along closed orbits, give rise to the phenomenon of an “orbital” magnetic moment oriented perpendicular to the plane of the cyclic motion. To a large extent, this moment is screened by the atomically localized part of the electron density. In addition to the most obvious cyclic motion performed by a molecule, namely, its free rotation in space, molecular vibrations in the form of pseudorotations can also lead to circular motions of certain atoms. A simple and particularly insightful example featuring such a pseudorotation is the Na_3_ molecule in its Jahn–Teller distorted 1^2^E′ ground state,^41,42^ where spectroscopic consequences have been discussed in the greatest detail within the context of a “molecular” Berry phase.^43^

It is our aim to provide a suitable methodology for the analysis of magnetic fields arising from such combined vibrational excitations. While rotational *g*-factors and spin-rotation coupling matrices have been thoroughly discussed in the literature,^44−47^ the investigation of vibrational and pseudorotational couplings has been restricted, so far, to *g*-factors only.^40^ In this section, for the sake of a transparent discussion, we provide a short derivation of rotational and vibrational couplings. A more detailed derivation can be found in the Supporting Information.

Considering the effect of nuclear rotation and vibration, a complete description of nuclear motion is possible via center of mass coordinates, Euler angles, and vibrational coordinates evaluated at the minimum of a selected potential energy surface. After expressing the classical kinetic energy in these coordinates, one obtains a metric, which may be used to construct a corresponding quantum Hamiltonian for the entire molecular system by canonical quantization.^48,49^ This procedure yields the total Hamiltonian
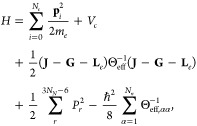
1where the first line corresponds to the electronic kinetic energy and all types of intramolecular Coulomb interactions, the second line corresponds to the rotational kinetic energy of the nuclear system in the respective coordinate frame, and the last line contains vibrational kinetic energy of the nuclei as well as a mass correction term, which is negligible. For small nuclear displacements, Θ_eff_ can be approximated by the nuclear inertia tensor Θ evaluated at the equilibrium geometry.

The nuclear rotational energy is relevant for vibrational and rotational coupling. Here, **J** = **L**_*e*_ + **L**_*N*_ + **G** denotes the total angular momentum, consisting of an electronic contribution , a nuclear contribution **L**_*N*_, and a vibrational angular momentum **G**. The latter is nonzero only for degenerate vibrational excitations and can be written as
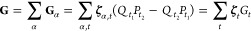
2with *t* indexing the doubly degenerate vibrational excitations and *Q*_*t*_1__ and *Q*_*t*_2__ denoting the respective normal coordinates. Furthermore, the scalar vibrational angular momentum *G*_*t*_ for each degenerate pair of eigenmodes has been introduced as well as the contribution of a single nucleus, **G**_α_. ζ_*t*_ denotes the so-called Coriolis coupling constant and  denotes the respective contribution of nucleus α, with  referring to the normalized, mass weighted displacement vector of the *i*-th vibrational mode.

We can perform first-order state correction with perturbation −(**J** – **G**)Θ^–1^**L**_*e*_ for the electronic system to obtain
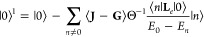
3The expression ⟨**J** – **G**⟩ denotes the expectation value with respect to the nuclear system. Usually, however, this term is treated classically. The electronic contribution to rotational and vibrational coupling parameters can then be calculated by evaluating the expectation value of a suitable interaction Hamiltonian in the perturbed state.

In order to obtain rotational and vibrational *g*-factors, we consider the paramagnetic interaction Hamiltonian with an external field . Note that **L**_α_ can be expressed as
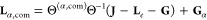
4with 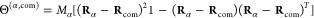 as the inertia tensor of nucleus α with respect to the center of mass. Using this and taking the expectation value in the perturbed state, this yields the rotationally and vibrationally induced magnetic moment  under the assumption of a Σ electronic ground state. The rotational and vibrational *g*-factors are
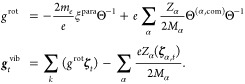
5with ξ^para^ as the paramagnetic magnetizability. Equation 5 agrees with the known expressions of vibrational and rotational *g*-factors from the literature.^40,47^ The magnetic moment, however, does not contain information about the magnetic field distribution within the molecule. Hence, to obtain measurable quantities, we calculate rotationally and vibrationally induced NMR splitting for each nucleus. The corresponding interaction Hamiltonian
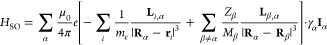
6is a spin–orbit coupling Hamiltonian with **L**_*i*,α_ as the electron angular momentum with respect to nucleus α, **L**_β,α_ as the nuclear angular momentum of nucleus β with respect to nucleus α, the nuclear gyromagnetic ratio γ_α_, and the nuclear spin operator **I**_α_. Calculating the expectation value in the perturbed state yields the rotationally and vibrationally induced magnetic field at nucleus α
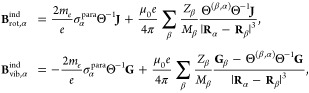
7with Θ^(β,α)^ denoting the inertia tensor of nucleus β with respect to nucleus α and  denoting the paramagnetic shielding tensor at nucleus α. For a rotationally induced magnetic field, this is consistent with ref (47) but generalizes the known result to the case of pseudorotational excitations.

### 2.2 Pseudorotation in the Bulk

In solid-state physics, the literature on an equivalent phenomenon speaks of ‘dynamical multiferroicity’ or a “phonon Zeeman effect”.^37−39^ Typically, magnetic moments in bulk structures are calculated from phonon modes and Born effective charge tensors. Significant effects are predicted for large charges in combination with small reduced masses, e.g., in hydride compounds such as CsH or CuH. Juraschek et al. investigated the possibility of inducing magnetization in spinless bulk materials by time-dependent electric polarization.^37^ Interested in the effective magnetic moment *M*, they calculate the contributions of all atoms in the unit cell by linking their rotational movement to a “phonon” angular momentum *L*. Assuming that only two phonon modes 1 and 2 are contributing, they find
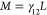
8with γ_12_ denoting the gyromagnetic ratio, and derive the latter from the circular motions of effectively charged nuclei α via
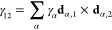
9using the same nomenclature as that introduced in Section 2.1 to distinguish the two orthogonal modes of a doubly degenerate vibration. The gyromagnetic ratio for each atom α is given by
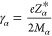
10with *Z*_α_^*^ denoting the Born effective charge obtained from DFT calculations. However, while this technique may provide useful predictions for macroscopic magnetic properties of bulk materials, a suitable description of magnetic field configurations on the subnanometer level can only be based on an analysis including electron density fluctuations upon vibrational excitation. This intrinsic limitation is addressed in Section 3.5.

### 2.3 Optical Excitation of a Rotating Electric Dipole

The dependence of the magnitude of the magnetic moment on the quadratic distance of a charge from its rotational axis, as can be seen, e.g., in the nuclear contribution to the *g*-factor in eq 5, clearly reminds of the classical definition of the magnetic moment
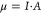
11if we interpret the rotating charges as a classical, closed loop of current *I* and area *A*. Assuming an opposite charge, but equal in magnitude, placed at the rotational center, it is tempting to imagine the charge dynamics during a pseudorotation as a rotating electric dipole vector. Indeed, as is shown below, the effective electric dipole moment of the phthalocyanines shows the behavior of a rotating vector during pseudorotation. However, the motion of the electric dipole moment μ_el_, an expectation value of the molecular system at a given geometry, does not directly translate into a magnetic moment for several reasons, as is discussed in the Results and Discussion section below.

Juraschek et al. further suggested to stimulate degenerate pairs of phonon modes via optical excitation, which would, in the case described above, lead to a magnetization that is proportional to the phonon frequency, the amplitude of the motion, and the phase relation between the two degenerate modes.^37^ Within the harmonic approximation, each of them, denoted for convenience as the *Q*_1_ and *Q*_2_ modes, will drive a periodic modulation of the molecular electric dipole moment, e.g., in either the *x* or *y* direction, respectively. This modulation will also be harmonic in character. Assuming a simultaneous excitation of the two planar modes, albeit with a phase difference of ± π/2, leads to the picture of the dipole moment vector p⃗ that rotates in that plane
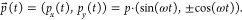
12Although the picture of a rotating electric dipole moment is too crude when discussing actual magnetic field geometries at the nanoscale, it has proven to be a suitable model for dynamical studies of the macroscopic magnetization in the bulk as a response to pulsed IR light. Note that irrespective of its limited value for the actual characterization of molecular magnetic fields, the presence of an electric dipole moment upon vibrational excitation is a necessary prerequisite for the suggested optical stimulation of vibrationally induced magnetism.

### 2.4 Computational Details

Electronic structure calculations for the moderately sized phthalocyanine complexes are performed via DFT, employing the B97D functional, a 9-parameter, dispersion-corrected GGA functional,^50^ in combination with the def2-SVP split valence basis set.^51^ The Q-Chem program package is used for all DFT calculations on H_2_–Pc and Cu–Pc.^52^ This higher level treatment of the electronic structure is compared to the results obtained with GFN-*x*TB, a semiempirical tight binding model conceived by the Grimme group,^53,54^ and extended to other metal phatalocyanines (Cu–Pc, Ni–Pc, Co–Pc, and Fe–Pc) in the Supporting Information.

Rotational *g*-tensors are calculated via the density-fitted Hartree–Fock theory with gauge-including atomic orbitals (DF-HF-GIAO) as implemented in the Molpro package,^55−57^ which also allows for computations of chemical shielding tensors and magnetizabilities.^58−60^ To improve computational efficiency during tensor evaluations, basis set fitting is employed, and polarization functions are removed from Cu and H atoms.

An alternative treatment of magnetic moments caused by rotations and pseudorotations has been suggested by Ceseroli and Tosatti,^61^ which is based on Berry–Phase calculations around closed orbits in the configuration space. For the sake of a direct comparison to previous studies on vibrationally induced molecular magnetism in smaller molecules, a selection of benzene and methane derivatives is discussed at the same level of theory.

## 3 Results and Discussion

### 3.1 Identification of IR-Active Pseudorotations

In the first step, the minimum energy structures of six variants of phthalocyanine (H_2_–Pc, Mn–Pc, Fe–Pc, Co–Pc, Ni–Pc, and Cu–Pc) as well as the benzene and methane derivatives are identified via DFT and a tight-binding ansatz as described above. The Hessians are evaluated at the same level of theory to obtain vibrational energies and their corresponding eigenmodes via matrix diagonalization. Structural information and detailed results are provided in the Supporting Information, which documents an acceptable performance of the GFN-*x*TB ansatz at a fraction of the computational effort of a DFT treatment. While vibrational energies deviate by less than 10% between the two approaches, larger deviations in terms of intensities are observed as they are known from more comprehensive studies.^62^ However, correct identification of the relevant doubly degenerate vibrational modes is possible also at the GFN-*x*TB level of theory, making it a viable alternative for larger structures in the future.

**Figure 2:**
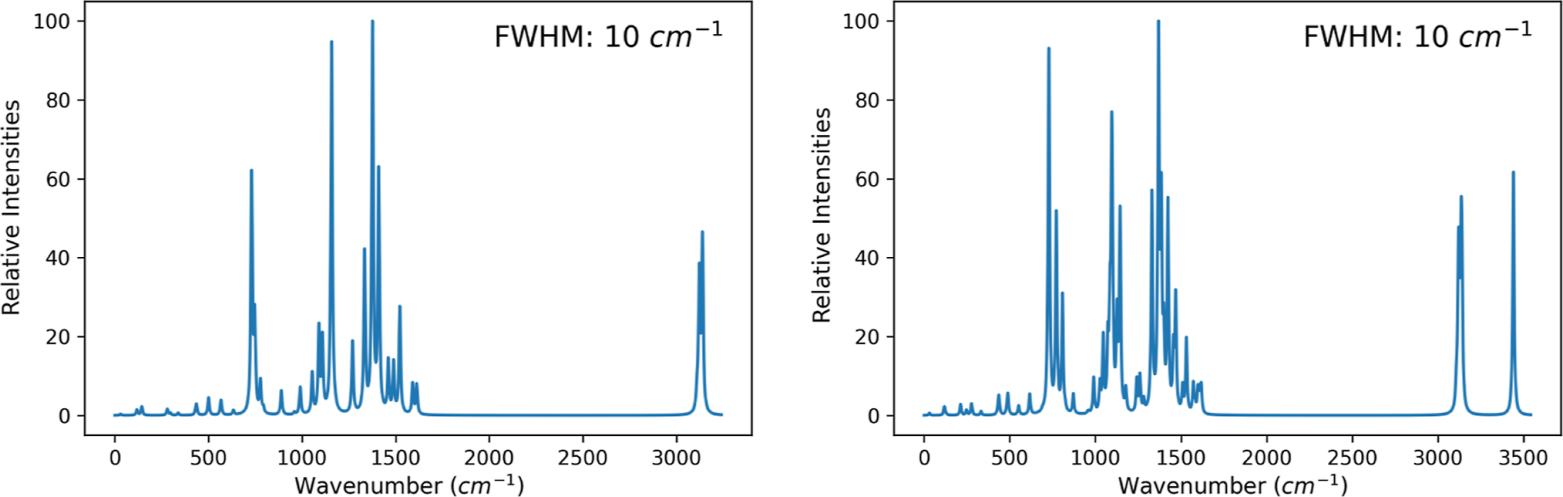
IR adsorption spectra of CuPc (left) and H_2_Pc (right) as two representative phthalocyanines, obtained from DFT calculations. A Lorentzian line shape function with an fwhm of 10 cm^–1^ was applied. Vibrations above 3000 cm^–1^ correspond to motions of H atoms; the larger peaks around 1000 cm^–1^ are mostly planar motions of N and C atoms.

For H_2_Pc and CuPc, the corresponding calculated IR adsorption spectra are plotted in Figure 2 as a representative of the whole group. The obtained spectra are in excellent agreement with experimental data;^63^ see the Supporting Information for a detailed comparison and spectral data of the other species.

In both cases, the spectrum can be broken into two parts, a lower part around 1000 cm^–1^, which is mostly due to planar motion of the nitrogen or carbon atoms, and an upper part around 3100 cm^–1^, which corresponds to planar motions of the hydrogen atoms of the benzene moieties. Qualitatively, but also in terms of line positions, the latter part of the spectrum is very closely related to the degenerate pairs of symmetric and antisymmetric C–H stretching vibrations of benzene (located at 3112 and 3125 cm^–1^ at the same level of theory, respectively). The large peaks clearly correspond to doubly degenerate vibrations or pseudorotations, except for the peak near 700 cm^–1^, which is the result of a nondegenerate out-of-plane motion of the N atoms. The additional peak at 3400 cm^–1^ in the case of H_2_Pc is unique in the series as it corresponds to the motion of the two H atoms at the center of the structure.

### 3.2 Rotational *g*-Factors Evaluation and Benchmarking

**Figure 3:**
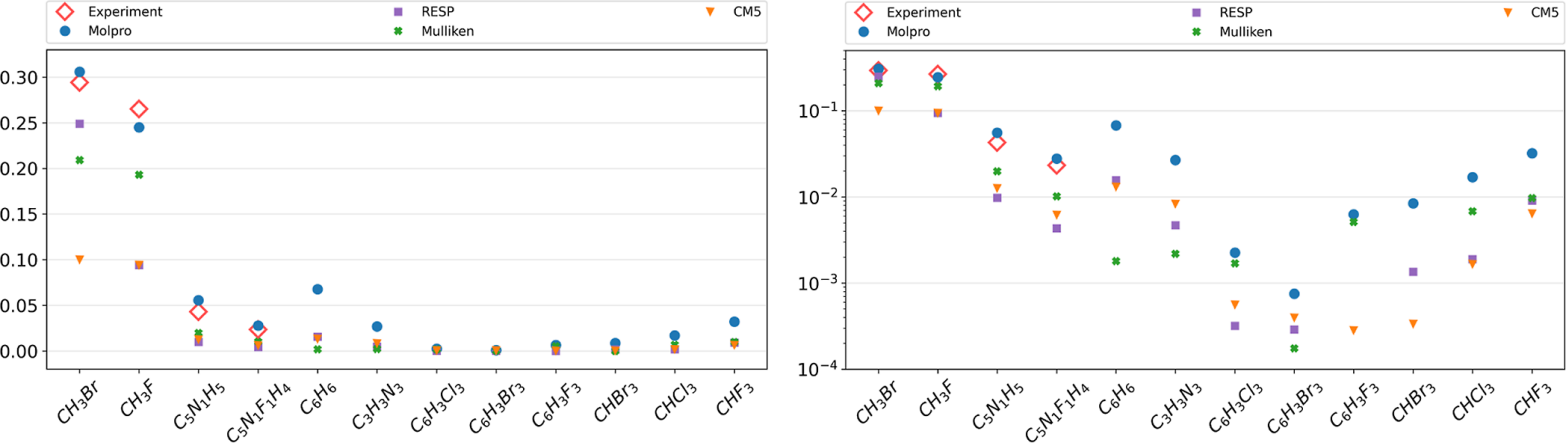
Absolute values of calculated and measured rotational *g*-factors, given in units of the nuclear magneton on linear (left) and logarithmic (right) scales. Theoretical values have been obtained with the Molpro via density-fitted Hartree–Fock theory with gauge-including atomic orbitals or by assuming a rigid rotation of various partial charges (RESP, CM5, and Mulliken) assigned to each atomic site.

We start with a series of benchmark calculations for experimentally and theoretically studied benzene and methane derivatives. Our results are summarized in Figure 3. Results obtained with Molpro show an average deviation of less than 10% from the experimental values known for the three components of the rotational g tensor, which documents sufficient accuracy for the purpose of this article.^44,64,65^

In the next step, we evaluate the rotational *g*-factors of H_2_Pc and CuPc for a rotation along the *z*-axis, i.e., perpendicular to the molecular plane, as they are a necessary ingredient for the evaluation of *g*_vib_ according to the method of Moss and Perry. Interestingly, rounded to the accuracy mentioned above, identical values of *g*_rot_ = 0.015 are obtained for both of them, which indicates a negligible variation over the whole series of metal atoms at this level of theory. On the other hand, a marginal impact of the central metal atom on the rotational *g*-factor is not fully unexpected, given the fact that it lies exactly on the rotational axis.

### 3.3 Vibrational *g*-Factors

After the calculation of rotational *g*-factors and the diagonalization of the molecular Hamiltonian, vibrational *g*-factors can be obtained either from eq 5, following the derivation of Moss and Perry, or via eq 10 with the help of fractional charges. While the choice of Born effective charges is straightforward from a solid state perspective, where a suitable description of effective macroscopic magnetic fields of bulk structures is desired, a correct treatment of intramolecular field geometries might necessitate a different approach. In the first step, we keep the concept of representative point charges but apply different charge localization strategies to the molecular benchmark set. We start with charges derived from the atomic polar tensor (APT), the molecular analogue to the Born effective charge tensor. As suggested by Milani et al.,^66^ only the *z*-component of the APT is used. In addition, partial charges derived from the electron density^67^ (Charge model 5, denoted as ‘CM5′ in the legend), from fits of the electrostatic potential^68^ (“RESP”), as well as standard Mulliken charges are used as input in eq 10. Other partial charges, e.g., derived from the APT such as GAPT charges,^69^ have been tested as well but provide inferior results and are therefore not discussed any further.

**Figure 4:**
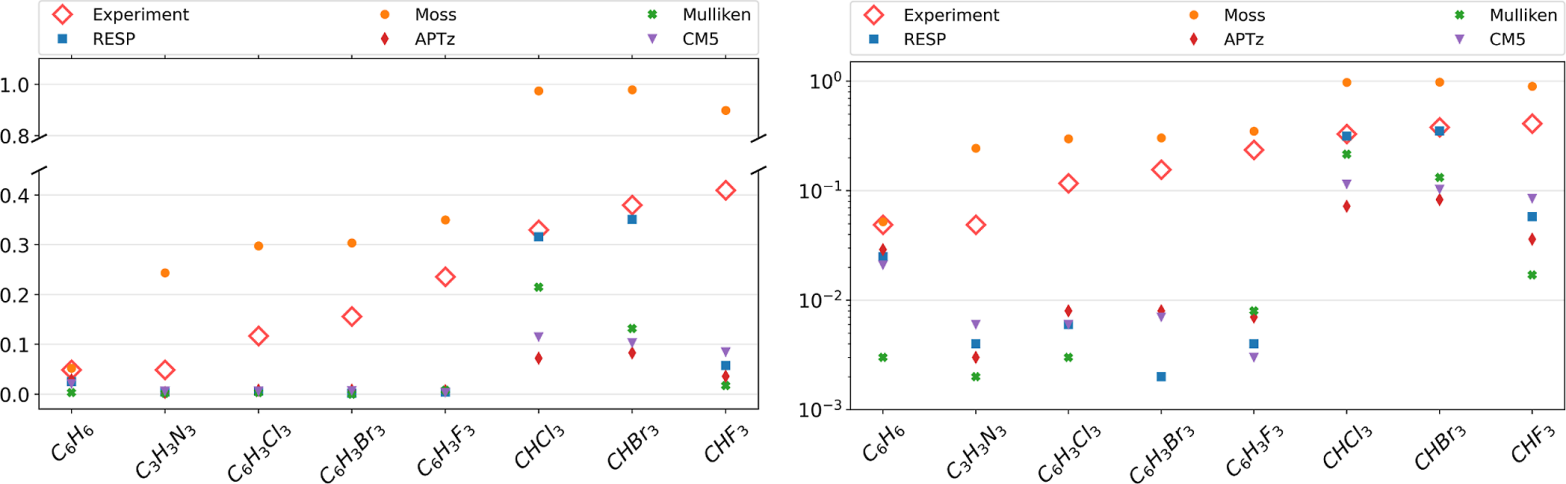
Absolute values of vibrational *g*-factors, obtained with all methods described in the text and compared to the experimentally confirmed eigenmodes at the following positions (from left to right, in cm^–1^): 1480, 1545, 1565, 1550, 1610, 1213, 1141, and 1371. The figures show linear (left) and logarithmic (right) scales. Experimental results are extracted from refs (65and, 70−72).

Our results are summarized in Figure 4. As becomes obvious from this comparison, all methods based on partial charges underestimate the vibrational *g*-factor substantially and are not even able to reproduce trends in the experimental data. A reason for this discrepancy may be an oversimplification that is intrinsic to all fractional charge models, namely, the assumption of point-like charge concentrations, a drastic simplification, even within a classical treatment, given the continuous character of the electron density distribution. For the sake of completeness, all models based on partial charges have been tested for their ability to predict rotational *g*-factors as well (see Figure 3), also without any success.

The ansatz of Moss and Perry, on the other hand, provides a fair reproduction of trends, although the results are still far away from quantitative agreement, in particular for the methane derivatives in the test set. Like all other methods mentioned above, their model also does not account for the substantial changes in the electron density that appear during molecular vibration (see later sections). However, a simple scaling by a factor of 0.404 reduces the relative root-mean-square deviation of *g*_vib_ to 25%; see the Supporting Information for details.

After this comparison of theoretical models to known benchmark sets that offer, at least for some systems, also experimental verification, we switch back to the discussion of phthalocyanines. Our findings are summarized in Table 1, which contains energies, relative intensities, electric dipole moments, magnetic dipole moments, and vibrational *g*-factors, calculated with the method of Moss and Perry, for the most promising pseudorotations. Due to intrinsic numerical inaccuracies, some of the modes are not perfectly degenerate and show minimal deviations in the range of a few wavenumbers. In these cases, the average is listed in the table. Mode indices refer to the energetic order from the lowest to highest. Note that in the case of H_2_Pc, vibrational degeneracies are only approximate due to the slightly reduced molecular symmetry, and both eigenmodes may have largely different intensities. Therefore, the *g*-factors are not calculated for H_2_Pc. Entries for μ_el_, the electric dipole moment, have been evaluated at an amplitude of , which corresponds to the standard deviation of a vibrational mode in its first excited state. The values of μ_mag_ have been calculated assuming an excitation to the first vibrationally excited state, i.e., ⟨*G*_*t*_⟩ = ℏ.

**Table 1:** Selected Pseudorotations of the Cu Phthalocyanine Obtained from DFT Calculations: Index (Sorted by Energy, the Lowest to Highest), Vibrational Frequency, Relative Intensity, Electric Dipole Moment, Magnetic Dipole Moment, *g*-Factor, and Induced Magnetic Moment of the Doubly Degenerate Vibrations^a^

index	ω/cm^–1^	rel. int.	μ_el_/Debye	μ_mag_/MHz/T	*g*_vib_
C_6_H_6_					
3, 4	605	0	0.000	1.56	0.205
21, 22	1467	5	0.069	0.39	0.052
CuPc					
79, 80	891	6	0.110	1.17	0.135
107, 108	1160	96	0.378	0.50	0.066
122, 123	1379	100	0.251	0.67	0.089
138, 139	1526	27	0.176	1.40	0.184
163, 164	3144	38	0.145	0.01	0.001

a For the magnetic moment, an excitation to the first excited state is assumed. Pseudorations of benzene are listed as well for comparison.

The non-IR-active pseudorotation of benzene (experimentally found at 606 cm^–1^, see ref (46)), picked by Ceresoli and Tosatti,^61^ is also included in the table for the sake of a direct comparison. In their work, a value of 0.79 is mentioned, evaluated via the Berry–Phase technique in a plane wave formalism using pseudopotentials. We note that this value, one of the very few estimates of *g*_vib_ available in the literature, differs from our value calculated via eq 5. However, it is almost reproduced if a factor of  is added to the Coriolis coupling constant, which might be due to an incorrect normalization of the corresponding eigenmodes used in ref (61). Unfortunately, there is no established standard of eigenmode normalization in current computational chemistry codes since actual amplitudes are seldom of interest, and either type may be found in the standard output of common packages.

### 3.4 Electric Dipole Moment Dynamics

**Figure 5:**
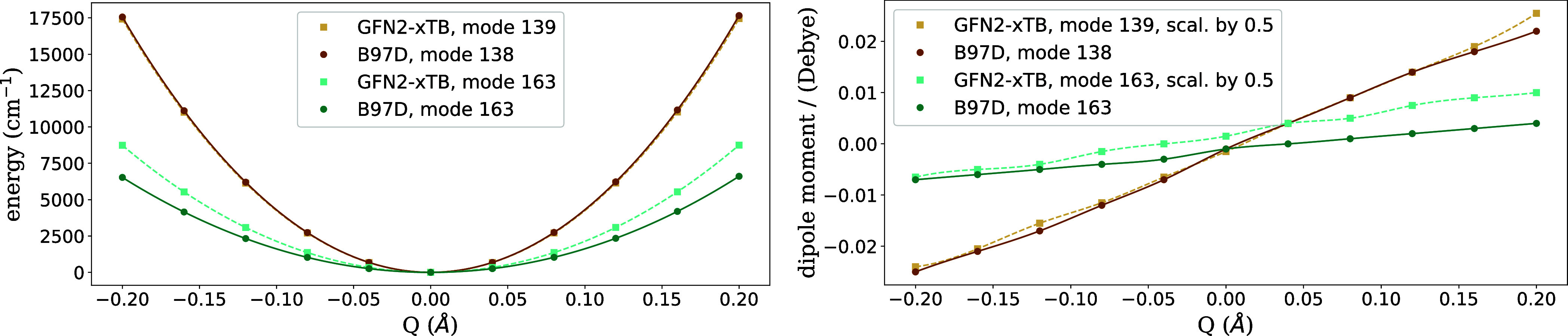
Scan over the energy (left) and the electric dipole moment (right) of CuPc as a function of the vibrational normal coordinate *Q*, corresponding to vibrational excitations at 1526 and 3144 cm^–1^, calculated with DFT as well as the GFN2-*x*TB tight binding ansatz. The mode index mentioned in the legend refers to the energetic order after diagonalization of the corresponding Hessian matrix.

Having investigated vibrational *g*-factors derived from either the vector coupling model of Moss and Perry or the more intuitive, yet qualitatively less applicable point charge models, we now look at vibrationally excited phthalocyanines from the perspective of electric dipole dynamics. For symmetry reasons, the molecule does not possess an electric dipole moment at the equilibrium geometry, but a distortion along a suitable, single vibrational eigenmode introduces a nonvanishing electric dipole moment. This can, e.g., be seen for the doubly degenerate vibrational excitations of CuPc at 1526 and 3144 cm^–1^ shown in Figure 5, where energy and dipole moment predictions obtained with DFT and the GFN2-*x*TB tight-binding model are compared.

Both quantities are plotted as a function of one of the two corresponding (degenerate) normal modes. The degenerate mode at 1526 cm^–1^ mostly corresponds to the displacement of N atoms and the other corresponds to the motion of H atoms, with reduced masses of around 12.4 and 1.1 amu, respectively. The latter explains the appearance of a wider parabola for the mode at higher energy. Note the remarkable agreement of the potential energy between both methods, which is almost perfect for the mode at 1526 cm^–1^. Regarding the dipole moment, an almost linear behavior is found for both methods for small deviations from the equilibrium geometry. Interestingly, the dipole moment obtained with the GFN2-*x*TB method deviates almost exactly by a factor of 2 from the DFT result.

**Figure 6:**
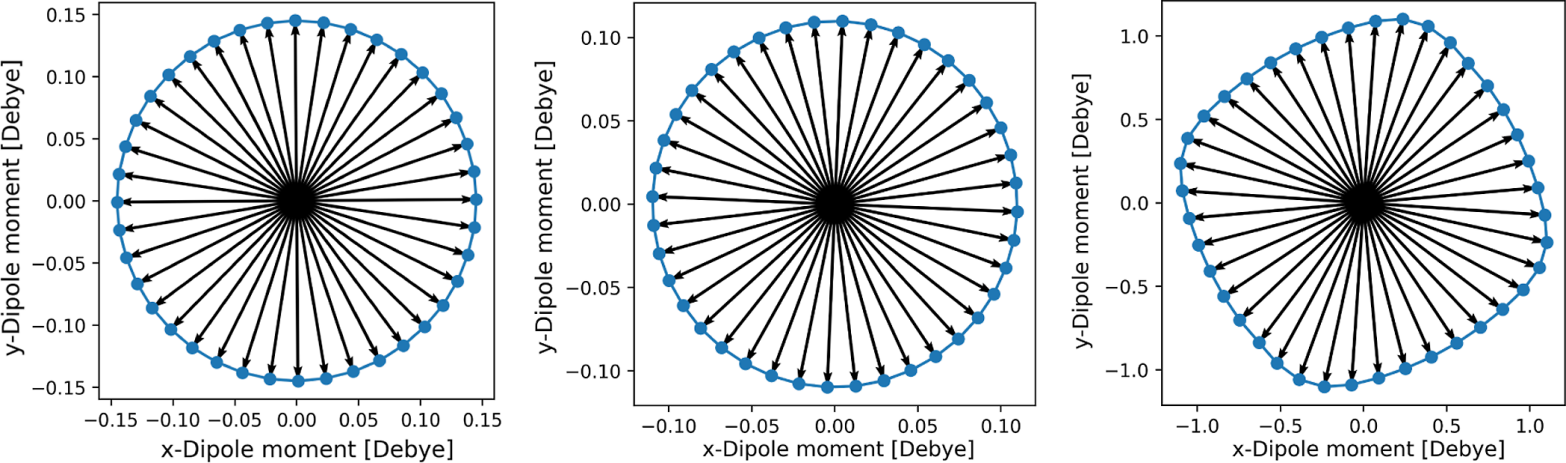
Plots of the electric dipole moment vector for pseudorotations at 3144 (left) and 891 cm^–1^ (center), obtained by displacing all atoms according to each pair of degenerate, normalized eigenmodes  via  with  and  as the eigenvectors, in steps of δϕ = π/20. Vibrational amplitudes of Δ*Q* = 0.074 Å and Δ*Q* = 0.077 Å have been chosen, respectively, corresponding to the standard deviation of each first vibrationally excited state. For comparison, the pseudorotation at 891 cm^–1^ is also presented at a much larger amplitude of Δ*Q* = 1 Å (right) to indicate the onset of anharmonic behavior in the dipole moment.

**Figure 7:**
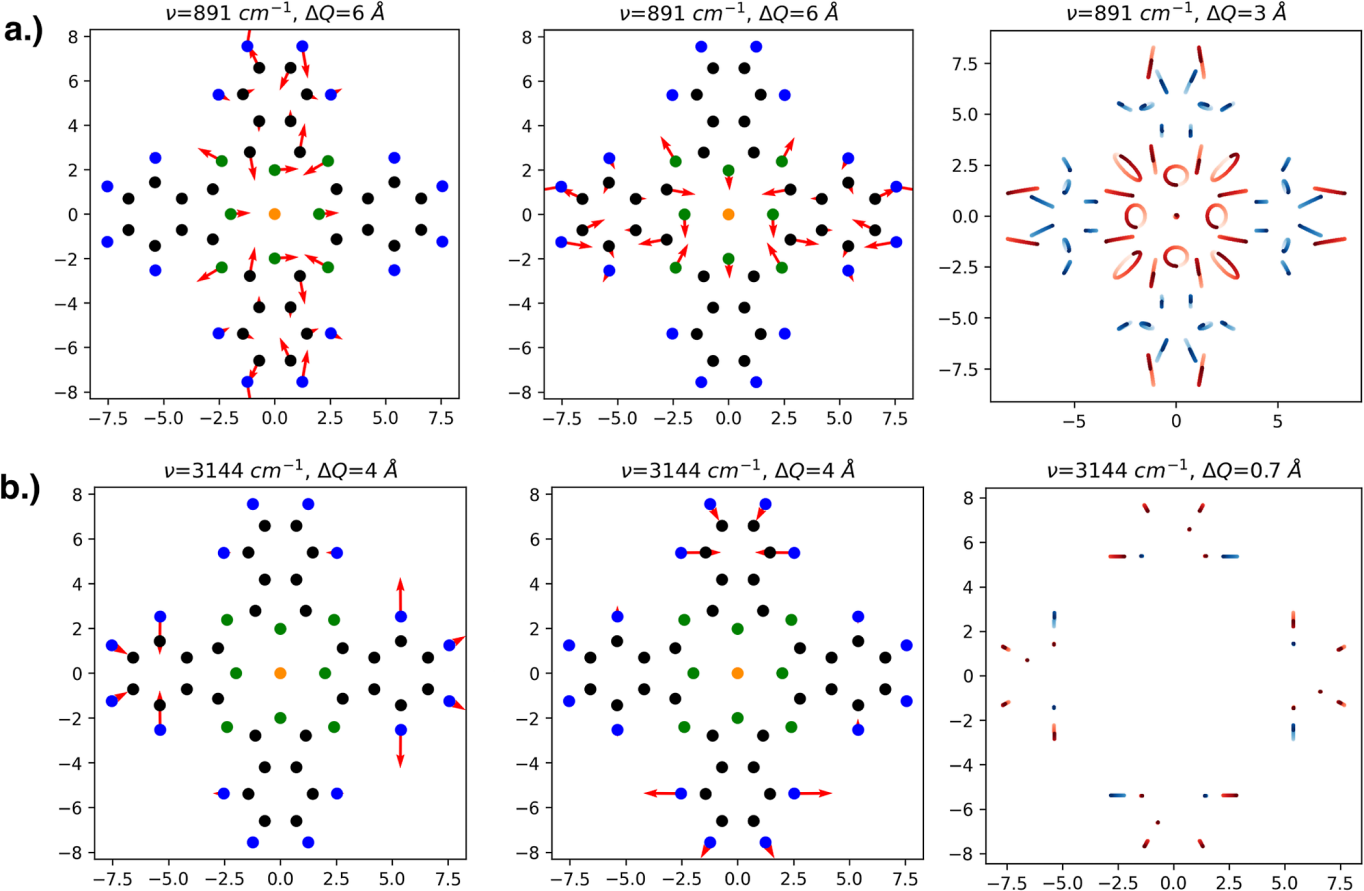
Pseudorotations of CuPc, realized by a linear combination of vibrational eigenmodes at (a) 891 and (b) 3144 cm^–1^. The first and second columns show the two modes and the third column shows the corresponding pseudorotation. Displacements are indicated by red arrows; right and left circularly moving atoms are indicated by red and blue trajectories, respectively. Exaggerated vibrational amplitudes Δ*Q* were chosen for better illustration. The vibration at lower energy mostly consists of collective motions involving all atoms, while the selected pseudorotation at higher energy represents mostly C–H stretching in the benzene moieties. Qualitatively, this description is the same for all phthalocyanines under study.

Combining two degenerate vibrational modes of CuPc with a phase shift of π/2, the linear behavior observed in Figure 5 must lead to a circular motion of the total molecular dipole moment, with its angular velocity determined by the frequency of the two degenerate eigenmodes; see the Supporting Information for further details. An example is displayed in Figure 6, which illustrates the degenerate modes of CuPc at 891 and 3144 cm^–1^. However, note that this (for small deviations from the equilibrium geometry) almost perfectly circular motion of the molecular electric dipole moment does not necessarily translate into circular motions of individual atoms, as can be seen from Figure 7, where the degenerate normal modes are projected back onto the motion of each atom. These two examples of a pseudorotation in phthalocyanines are significantly different in character: One corresponds to a combined motion of all atoms and contains several circular trajectories, while the other corresponds mostly to C–H stretching in the benzene moieties and essentially linear nuclear trajectories.

It is equally problematic to state the reverse argument, namely, that the generation of a magnetic moment is hinged on a rotating electric dipole: For example, the pseudorotation mentioned above, selected by Ceresoli and Tosatti,^61^ yields a nonzero vibrational *g*-factor, although it is based on two degenerate modes that are not IR-active. Introducing an evaluation method based on the Berry phase, they reveal a surprisingly low screening of the rotating ions by the electrons despite the aromaticity of the molecule, and a more complex behavior of the electric dipole moment is predicted. We will return to these earlier findings when discussing electron density fluctuations upon pseudorotation. Furthermore, even if an electric dipole moment exists and is only changing in orientation, the magnetic moment generated by the rotating centers of positive and negative charges will depend on their actual distance from the center of mass and the actual amount of fractional charge, none of which are unambiguously retrievable from the electric dipole moment alone. Plots of the motion of the two centers of charge during selected pseudororations are included in the Supporting Information, revealing that a dipole vector constructed from these two central moments is also performing a circular translation besides an actual rotation.

All of these findings point in the same direction: Local molecular magnetic fields, created through excitation of vibrational modes, are badly characterized by a single “vibrational *g*-factor” in the first place as the corresponding magnetic field geometry may be far too complex to be well described by a simple dipole term. As a consequence, vibrationally induced hyperfine splittings as well as chemical shifts, even those of identical nuclei, will differ with respect to the position of each nucleus within the same molecule. We note that these findings represent an analogy to atomic site-dependent *g*-factors as they are observable in the case of conventional Zeeman splitting, caused by an obviously inhomogeneous electron spin density of an open-shell molecule.^73^ Interestingly, the latter had been proven in 2015 on Mn phthalocyanine, yet another member of the phthalocyanine family, but of quartet multiplicity.

### 3.5 Electron Density Dynamics

**Figure 8:**
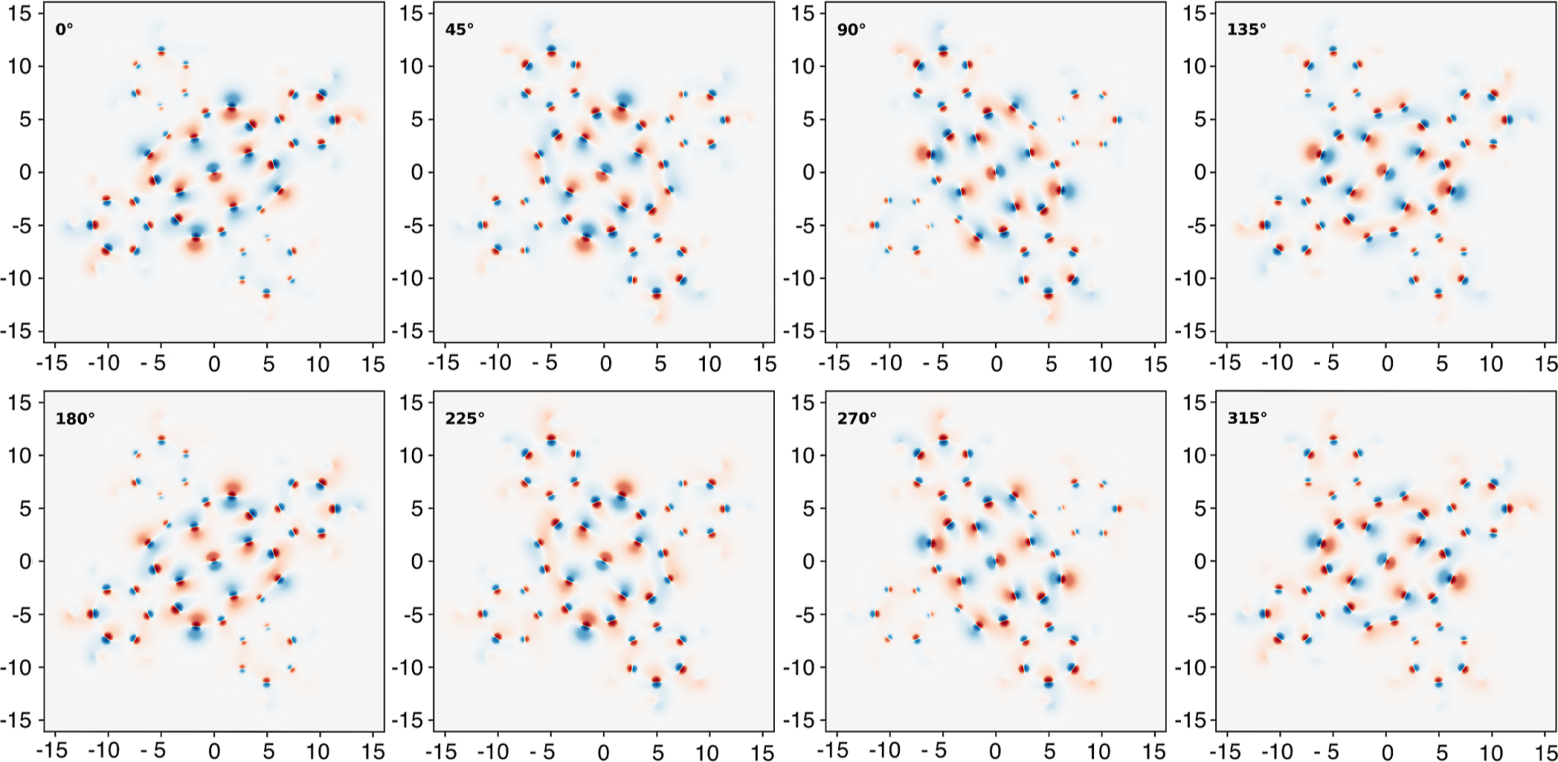
Changes in the electron density distribution of CuPc upon pseudorotation at 1526 cm^–1^, in steps of 45 degrees and with amplitude Δ*Q* according to the standard deviation of the first excited state, made visible through a projection onto the molecular plane. Positions are given in Bohr; the electron density has been plotted logarithmically for a meaningful illustration of electron charge displacement.

**Figure 9:**
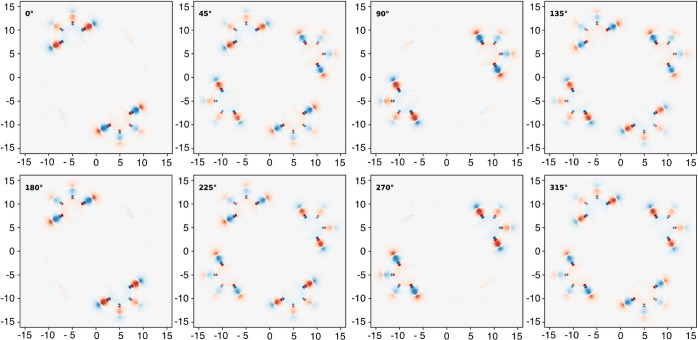
Changes in the electron density distribution of CuPc upon pseudorotation at 3144 cm^–1^, in steps of 45 degrees and with amplitude Δ*Q* according to the standard deviation of the first excited state, made visible through a projection onto the molecular plane. Positions are given in Bohr; the electron density has been plotted logarithmically for a meaningful illustration of electron charge displacement.

A last investigation of electric charge dynamics, this time with the highest possible resolution, brings us to the discussion of electron density fluctuations upon pseudorotation. For two degenerate modes, at 1526 and at 3144 cm^–1^, changes of the electron density distribution upon vibrational excitation, evaluated with DFT using the B97D functional, are plotted in Figures 8 and 9. Again, a vibrational amplitude of  has been chosen for display. An excess or lack of electron density in comparison to the unperturbed density at the equilibrium geometry is printed in blue or red, respectively. The electron density has been integrated in the *z*-direction, so the plots can be understood as projections of the electron density onto the molecular plane. In order to visualize the minute differences in the overall charge distribution, the density is plotted on a logarithmic scale. Otherwise, the substantial relocalization of atom-centered electron charge densities (“core” electrons) in the course of atomic displacements would have hampered the readability of the illustrations.

From Figure 8, it becomes immediately obvious that pseudorotations in macrocycles do cause periodic, but complicated patterns of charge displacements, which confirms to some extent the findings of ref (61) for benzene. In the case of the pseudorotation of CuPc at about 1526 cm^–1^, where mostly C and N atoms are involved, the charge perturbations along the conjugated double bonds of the inner rings are dominating but far too complex to be approximated by a single rotating dipole. Instead, the electron density is constantly rotating along with the displaced atoms. The behavior of the electron density is particularly interesting in regions near the metal center: an almost perfectly circular motion of electron density is driven by the periodic vibration of the central atom. However, this motion of the central metal atom is present in only a few eigenmodes, as can be seen by comparing Figures 8 and 9. For the pseudorotation at about 3144 cm^–1^, the electron density is modulated only at the hydrogen atoms, which are showing an almost perfectly linear type of motion (see also Figure 7). According either to Moss and Perry or to the “multiferroism” ansatz of Juraschek, linear motions do produce a vibrational *g*-factor of 0 since the cross product of the eigenvectors vanishes. As has been indicated already before, this is in harsh contrast to the oversimplified but intuitive interpretation of a (confirmed) rotating electric dipole moment as the generator of a molecule-wide magnetic dipole field. A definitive answer regarding the actual geometry of this vibrationally induced magnetic field can only be provided by experiments, which brings us to the next section, discussing possible observables for measurements in the laboratory.

### 3.6 Vibrationally Detuned Chemical Shielding

If vibrationally induced magnetic fields, created via IR excitation of pairwise degenerate vibrational modes, are a physical reality, they should manifest themselves experimentally through comparably weak “vibrationally” induced splittings, e.g., in the hyperfine structure of the central metal atom. Of the centers investigated in this study, the stable and most abundant isotopes  would be suitable candidates, in principle, for measurements of an optically induced, “vibrational” hyperfine splitting due to their nonzero nuclear spins of 5/2, 7/2, and 3/2.

However, the accurate description and measurement of hyperfine splittings in molecules are highly challenging. Instead, we propose a much simpler experimental approach based on standard NMR measurements of chemical shifts: a vibrationally induced magnetic field, activated through the photoexcitation of a suitable, IR-active pseudorotation, should provoke a significant change in the chemical shift observed for the resonance of each atomic nucleus simply due to the superposition of the external and intramolecular magnetic fields. These shifts will be easily distinguishable from the well-known, minimal effects of vibrationally induced chemical shifts^74^ stemming directly from changes of the electron density distribution due to zero point vibrational energy or a certain Boltzmann occupation of vibrational levels at a given temperature. Contrary to the latter, effects due to the IR excitation of a suitable pseudorotation will be optically triggered and can cause additional chemical shifts in either direction, simply by changing the phase difference appearing in eq 12 from −π/2 to π/2.

**Figure 10:**
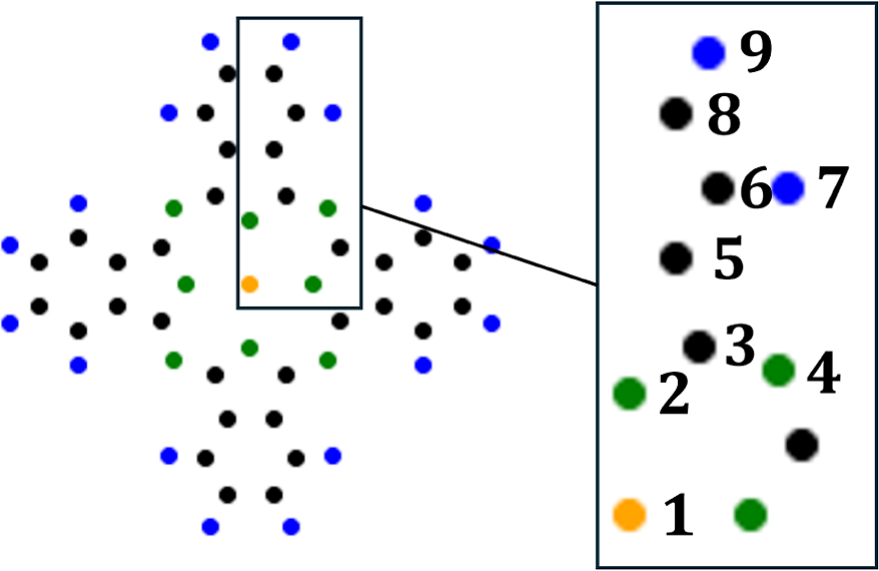
Positions of all distinguishable nuclei within CuPc, numbered according to their distance from the center; see Table 2 for the corresponding information on magnetic field strength.

In Table 2, we summarize the shifting effects caused by selected pseudorotations for all distinguishable nuclei in Cu phthalocyanine. Given the similarities in the vibrational modes as well as the vibrational *g*-factors found for other representatives, this table may serve as a first, crude reference for the whole class of metal phthalocyanines. The index corresponds to the distinguishable occurrences of each element within the molecule, ranked with respect to distance *d* from the center; see Figure 10. For N, H, and Cu, these values represent the vibrational chemical shift (as C does not possess a nuclear spin) at this external field strength and are thus measurable quantities. They are calculated for a vibrational excitation of ⟨*G*_*t*_⟩ = ℏ in the respective mode. Note that signs can be positive or negative, depending on the actual orientation of the pseudorotation, but the relative sign between shifts stays the same. For the degenerate vibrations at 3144 cm^–1^, the induced chemical shift is (similar to the corresponding vibrational *g*-factor) approximately 2 orders of magnitude smaller than that for the degenerate vibrations at 1526 cm^–1^.

**Table 2:** Estimates of the Magnetic Field Induced by a Stimulated Pseudorotation of CuPc at 1526 cm^–1^, with ⟨*G*_*t*_⟩ = ℏ, Given for the *z*-Direction, Evaluated at Each Distinguishable Nucleus, and Expressed as Chemical Shifts, i.e., in ppm Relative to an External Field *B*_0_ = 1 T; See Figure 10 for Positional Information

index	element	*B*_vib_/*B*_0_	*d*/Å
1	Cu	5.3	0.0
2	N	11.9	2.0
3	C	10.8	3.0
4	N	9.5	3.4
5	C	5.8	4.3
6	C	0.7	5.6
7	H	–0.3	5.9
8	C	–0.5	6.6
9	H	1.0	7.7

## 4 Conclusions

Phthalocyanines, a highly versatile group of aromatic planar macrocyclic molecules, are well-known and appreciated for their magnetic properties. However, previous studies have been dedicated almost exclusively to spin Zeeman effects. In this study, the simultaneous, concerted excitation of two degenerate vibrational modes of a phthalocyanine molecule has been presented as a tool to create a localized, “molecular” magnetic field. By means of a computational study employing DFT, suitable vibrational excitations have been identified, and known theoretical descriptions have been presented, tested, and extended in order to allow for predictions of magnetic dipole moments and changes in the shielding tensors caused by these concerted vibrations.

Interestingly, although some IR-active pseudorotations do feature a rotating electric dipole moment, a direct conversion of this motion into a magnetic dipole moment, as suggested by classical electrodynamics, is not applicable: fluctuations of the electron density, observed during pseudorotation, suggest a much more complex geometry of the magnetic field even in these cases and indicate that magnetic field effects, e.g., on the nuclear spins of a molecule, must be site-specific due to substantial field inhomogeneities.

Fortunately, more intricate theoretical models have been known for decades, stemming from the golden era of NMR spectroscopy, where the motions of all nuclei upon pseudorotation are tracked and translated into separate contributions to the magnetic dipole field. Here, the description of Moss and Perry from 1972 stands out, but a closely related, more pragmatic ansatz has been pushed forward by the solid state community, termed as the ‘dynamical multiferroic effect’, where electron motion is accounted for through an effective nuclear charge derived from the polarization tensor. Inspired by this approach, we employ its molecular analogue, the atomic polarization tensor, and test several alternative partial charge models, e.g., derived from the electron density or the Coulomb potential. However, while the original ansatz, combined with modern ab initio evaluations of vibrational eigenmodes and rotational *g*-factors, could be improved by a simple scaling, methods based on point charges do not agree with the experiment at all. Hence, further work is needed in terms of method development, as well as the generation of accurate experimental data. Nevertheless, applying our best model to the class of phthalocyanines, we make first predictions for changes in the chemical shifts upon photoexcitation of the most promising IR-active pseudorotations. Experimental detection of the latter should be straightforward as these shifts will be triggered by the optical excitation of suitable modes and their sign will be dependent on the phase angle between the two oscillations.

Although vibrational couplings to spin magnetism have been known for decades in microwave spectroscopy, our work proposes a connection between an optically active and therefore experimentally accessible vibrational excitation of a molecule and its magnetic field generated at specific sites; the predicted changes in magnetic shielding constants, if confirmed by experiment, can be interpreted as first measurements of a vibrationally induced magnetic field with intramolecular resolution. The ability to switch or change the direction of this molecular magnetic field by optical setups, in combination with the potential of thin film depositions of phthalocyanines, makes this group of molecules an exciting target of future studies, adding an entirely new facet to this branch of magnetochemistry.

## Supporting Information Available

Derivation of spin–vibration coupling parameters and rescaled *g*-factors, comparison to GFN2-*x*TB tight binding results, IR spectra for all phthalocyanines where experimental data are available, additional electron density plots, dipole moment scans along an IR-active normal mode, optimized geometries of all phthalocynanine structures in the *xyz*-format, and excerpts of Molpro output files containing information on vibrational modes (PDF)
