# Supplementary material for: Molecular Pseudorotation in Phthalocyanines as a Tool for Magnetic Field Control at the Nanoscale
Source: J Am Chem Soc. 2024 May 14;146(21):14620–32. doi: 10.1021/jacs.4c01915 (PMC11140746; doi:10.1021/jacs.4c01915)
Supplement: Supplementary file 1 — ja4c01915_si_001.pdf [file ja4c01915_si_001.pdf]

Supporting Information: Molecular pseudorotation in phthalocyanines as a tool for magnetic field control at the nanoscale

Raphael Wilhelmer, Matthias Diez, Johannes K. Krondorfer and Andreas W. Hauser*

Institute of Experimental Physics, Graz University of Technology, Petersgasse 16, A-8010 Graz, Austria. Phone: +43 (316) 873-8157, Fax: +43 (316) 873-108152

E-mail: andreas.w.hauser@gmail.com

In the first section of this Supporting Information, we provide the details on the calculation of vibrational g-factors and spin-vibration coupling using perturbation theory and the rescaled vibrational g-factors. Section S2 gives a summary of vibrational frequencies in tabulated form, obtained with the GFN-xTB tight binding ansatz of the Grimme group.[1,2] In Section S3, IR spectra are provided for all phthalocyanines, calculated with DFT via the Q-Chem program package,[3] and compared to the corresponding GFN-xTB results. Additional data, comparing the calculated to experimental values, is also presented. This is followed by a series of graphical illustrations of electron density changes in Cu phthalocyanine upon selected pseudorotations, evaluated with Q-Chem. In Section S5, dipole moment scans along a IR-active normal mode of H_2Pc are presented for a comparison between the two computational methods, i.e. the tight binding ansatz and the results obtained with dispersion-corrected DFT using the PBE functional. Additionally, the origin of the rotating dipole moment is illustrated via the center of charges and different partial charges are

investigated in terms of their ability to reproduce the electric dipole moment. Finally, in Section S6, optimized geometries of all phthalocynanine structures are listed in xyz-format, followed by excerpts of Q-Chem output files containing information on vibrational modes of CuPc evaluated with DFT in Section S7.

Contents

S1 Derivation of vibrational coupling parameters and rescaled g-factors 4

S1.1 Derivation of vibrational g-factors and spin-vibration coupling parameters . . 4

S1.2 Rescaled vibrational g-factors 7

S2 GFN2-xTB data for comparison 9

S3 IR spectra details 11

S4 Electron density plots 16

S5 Electric dipole moment 21

S5.1 H_2Pc dipole scans 21

S5.2 Rotating dipoles interpreted via the center of charge 21

S5.3 Rotating dipoles calculated from partial charges 24

S6 Optimized Geometries 26

S6.1 H_2Pc 26

S6.2 FePc 28

S6.3 CoPc 30

S6.4 NiPc 32

S6.5 CuPc 34

S7 Vibrational Eigenmodes 37

S7.1 891 cm^{-1} 37

S7.2 1160 cm^{-1} 39

S7.3 1336 cm^{-1} 42

S7.4 1526 cm^{-1} 44

S7.5 3144 cm^{-1} 46

References **49**

S1 Derivation of vibrational coupling parameters and rescaled g-factors

S1.1 Derivation of vibrational g-factors and spin-vibration coupling parameters

Considering the effect of nuclear rotation and vibration, a complete description of nuclear motion is possible via center of mass coordinates, Euler angles and vibrational coordinates evaluated at the minimum of a selected potential energy surface. After expressing the classical kinetic energy in these coordinates, one obtains a metric, which may be used to construct a corresponding quantum Hamiltonian for the entire molecular system by canonical quantization.[4,5] This procedure yields the total Hamiltonian

$$\begin{aligned} H = & \sum_{i=0}^{N_e} \frac{\mathbf{p}_i^2}{2m_e} + V_c \\ & + \frac{1}{2}(\mathbf{J} - \mathbf{G} - \mathbf{L}_e)\Theta_{\text{eff}}^{-1}(\mathbf{J} - \mathbf{G} - \mathbf{L}_e) \\ & + \frac{1}{2}\sum_{r}^{3N_N-6} P_r^2 - \frac{\hbar^2}{8}\sum_{\alpha=1}^{N_n}\Theta_{\text{eff},\alpha\alpha}^{-1}, \end{aligned} \tag{S1}$$

where the first line corresponds to the electronic kinetic energy and all types of intramolecular Coulomb interactions, the second line corresponds to the rotational kinetic energy of the nuclear system in the respective coordinate frame, and the last line contains vibrational kinetic energy of the nuclei and a mass correction term, which is negligible. For small displacements from the equilibrium Θ_{eff} can be approximated by the nuclear inertia tensor Θ in the equilibrium configuration.

The nuclear rotational energy is relevant for vibrational and rotational coupling. Here $\mathbf{J} = \mathbf{L}_e + \mathbf{L}_N + \mathbf{G}$ denotes the total angular momentum, with electronic contribution $\mathbf{L}_e = \sum_{i=0}^{N_e} \mathbf{L}_i$ and nuclear contribution $\mathbf{L}_N$. The vibrational angular momentum $\mathbf{G}$ is only non-

zero for degenerated vibrational excitation and can be written as

$$\mathbf{G} = \sum_{\alpha} \mathbf{G}_{\alpha} = \sum_{\alpha,t} \boldsymbol{\zeta}_{\alpha,t}(Q_{t_1}P_{t_2} - Q_{t_2}P_{t_1}) = \sum_{t} \boldsymbol{\zeta}_t G_t, \tag{S2}$$

with t indexing the doubly degenerate vibrational excitations and Q_{t_1}, Q_{t_2} denoting the respective normal coordinates. Furthermore, the scalar vibrational angular momentum G_t for each degenerate pair of eigenmodes has been introduced. $\boldsymbol{\zeta}_t$ denotes the so-called Coriolis coupling constant and $\boldsymbol{\zeta}_{\alpha,t} = \boldsymbol{d}_{\alpha,t_1} \times \boldsymbol{d}_{\alpha,t_2}$ the respective contribution of nucleus α, where $\boldsymbol{d}_{\alpha,t_i}$ denotes the normalized mass weighted displacement vector for vibrational mode i.

We can perform first order state correction with perturbation $-(\mathbf{J} - \mathbf{G})\Theta^{-1}\mathbf{L}_e$ for the electronic system to obtain

$$|0\rangle^1 = |0\rangle - \sum_{n \neq 0} \langle \mathbf{J} - \mathbf{G} \rangle \Theta^{-1} \frac{\langle n| \mathbf{L}_e |0\rangle}{E_0 - E_n} |n\rangle \,. \tag{S3}$$

The expression $\langle \mathbf{J} - \mathbf{G} \rangle$ denotes the expectation value with respect to the nuclear system. Usually, however, this term is treated classically. The electronic contribution to rotational and vibrational coupling parameters can now be calculated by evaluating the expectation value of a suitable interaction Hamiltonian in the perturbed state.

In order to obtain rotational and vibrational g-factors we consider the paramagnetic interaction Hamiltonian with an external field

$$H_B^{\text{para}} = \left(\frac{e}{2m_e} \mathbf{L}_e - \sum_{\alpha} \frac{Z_\alpha e}{2M_\alpha} \mathbf{L}_\alpha \right) \cdot \mathbf{B} \tag{S4}$$

. The angular momentum of nucleus α can be expressed as

$$\mathbf{L}_{\alpha,\text{com}} = \Theta^{(\alpha,\text{com})} \Theta^{-1} \left(\mathbf{J} - \mathbf{L}_e - \mathbf{G} \right) + \mathbf{G}_\alpha, \tag{S5}$$

with $\Theta^{(\alpha,\text{com})} = M_\alpha[(\mathbf{R}_\alpha - \mathbf{R}_{\text{com}})^2 \mathbb{1} - (\mathbf{R}_\alpha - \mathbf{R}_{\text{com}})(\mathbf{R}_\alpha - \mathbf{R}_{\text{com}})^T]$ the inertia tensor of nucleus

α with respect to the center of mass. Using this yields the electronic and nuclear contribution to the rotationally and vibrationally induced magnetic moment are

$$\mu_{e,i} = -\frac{e}{2m_e}\left[\langle 0|\, L_{e,i}\,|0\rangle - \sum_{k,l}(\Theta^{-1})_{kl}\langle J_k - G_k\rangle \sum_{n\neq 0}\frac{\langle 0|\, L_{e,i}\,|n\rangle \langle n|\, L_{e,l}\,|0\rangle + c.c.}{E_0 - E_n}\right] \tag{S6}$$

$$\boldsymbol{\mu}_N = e\sum_{\alpha}\frac{Z_\alpha}{2M_\alpha}\Theta^{(\alpha,\mathrm{com})}\Theta^{-1}(\mathbf{J} - \mathbf{L}_e - \mathbf{G}) + \sum_t\sum_\alpha \frac{eZ_\alpha \boldsymbol{\zeta_{\alpha,t}}}{2M_\alpha}G_t. \tag{S7}$$

For a Σ ground state, this can be written as $\boldsymbol{\mu}^{\mathrm{ind}} = g^{\mathrm{rot}}\mathbf{J} - \sum_t \boldsymbol{g}_t^{\mathrm{vib}} G_t$, with the rotational and vibrational g-factors

$$\begin{aligned} g^{\mathrm{rot}} &= -\frac{2m_e}{e}\xi^{\mathrm{para}}\Theta^{-1} + e\sum_\alpha \frac{Z_\alpha}{2M_\alpha}\Theta^{(\alpha,\mathrm{com})}\Theta^{-1} \\ \boldsymbol{g}_t^{\mathrm{vib}} &= \sum_k (g^{rot}\boldsymbol{\zeta}_t) - \sum_\alpha \frac{eZ_\alpha(\boldsymbol{\zeta}_{\alpha,t})}{2M_\alpha}. \end{aligned} \tag{S8}$$

with ξ^{para} the paramagnetic magnetizibility given by

$$\xi_{ij}^{\mathrm{para}} = -\left(\frac{e}{2m}\right)^2 \sum_{n\neq 0}\frac{1}{E_0 - E_n}\left(\langle 0|\, L_{e,j}\,|n\rangle \langle n|\, L_{e,i}\,|0\rangle + c.c.\right) \tag{S9}$$

Equation S8 reproduces the formulas of vibrational and rotational g-factors from literature.[6,7]

The magnetic moment, however, does not contain information on the magnetic field distribution within the molecule. To obtain measurable quantities we calculate the rotationally and vibrationally induced NMR splitting for each nucleus. The corresponding interaction Hamiltonian

$$H_{\mathrm{SO}} = \sum_\alpha \frac{\mu_0}{4\pi}e\left[-\sum_i \frac{1}{m_e}\frac{\mathbf{L}_{i,\alpha}}{|\mathbf{R}_\alpha - \mathbf{r}_i|^3} + \sum_{\beta\neq\alpha}\frac{Z_\beta}{M_\beta}\frac{\mathbf{L}_{\beta,\alpha}}{|\mathbf{R}_\alpha - \mathbf{R}_\beta|^3}\right]\cdot \gamma_\alpha \mathbf{I}_\alpha \tag{S10}$$

is the spin-orbit coupling Hamiltonian with $\mathbf{L}_{i,\alpha}$ the electron angular momentum with respect to nucleus α, $\mathbf{L}_{\beta,\alpha}$ the nuclear angular momentum of nucleus β with respect to nucleus α, the nuclear gyromagnetic ratio γ_α and the nuclear spin operator $\mathbf{I}_\alpha$. Analogously to the

calculation of the g-factor we calculate the expectation value in the perturbed state and use a similar expression for $\mathbf{L}_{\beta,\alpha}$ as in Equation S5. In a Σ ground state the rotationally and vibrationally induced magnetic field at nucleus α can then be written as

$$\begin{aligned}\mathbf{B}^{\text{ind}}_{\text{rot},\alpha} &= \frac{2m_e}{e}\sigma^{\text{para}}_{\alpha}\Theta^{-1}\mathbf{J} + \frac{\mu_0 e}{4\pi}\sum_{\beta}\frac{Z_\beta}{M_\beta}\frac{\Theta^{(\beta,\alpha)}\Theta^{-1}\mathbf{J}}{|\mathbf{R}_\alpha - \mathbf{R}_\beta|^3} \\ \mathbf{B}^{\text{ind}}_{\text{vib},\alpha} &= -\frac{2m_e}{e}\sigma^{\text{para}}_{\alpha}\Theta^{-1}\mathbf{G} + \frac{\mu_0 e}{4\pi}\sum_{\beta}\frac{Z_\beta}{M_\beta}\frac{\mathbf{G}_\beta - \Theta^{(\beta,\alpha)}\Theta^{-1}\mathbf{G}}{|\mathbf{R}_\alpha - \mathbf{R}_\beta|^3}\end{aligned} \tag{S11}$$

with $\Theta^{(\beta,\alpha)}$ the inertia tensor of nucleus β with respect to nucleus α and $\sigma^{\text{para}}_{\alpha}$ the paramagnetic shielding tensor at nucleus α given by

$$(\sigma^{\text{para}}_{\alpha})_{kj} = \frac{\mu_0}{4\pi}\frac{e^2}{2m_e^2}\sum_i\sum_n\frac{1}{E_0 - E_n}\left[\langle 0|\,\frac{(L_{i,\alpha})_k}{|\mathbf{R}_\alpha - \mathbf{r}_i|^3}\,|n\rangle\,\langle n|\,(L_{e,\alpha})_j\,|0\rangle + c.c.\right] \tag{S12}$$

For the rotationally induced magnetic field, this is consistent with Ref. 7 but generalizes the result for pseudorotational excitations.

S1.2 Rescaled vibrational g-factors

As explained in the main article, vibrational g-factors calculated by the method of Moss and Perry generally overestimate the vibrational g-factors. However, re-scaling the g-factors by a factor of 0.404 (which minimized the mean squared error of the g-factors to 0.05) leads to much better results. This can be seen in Figure S1, where the rescaled g-factors are plotted.

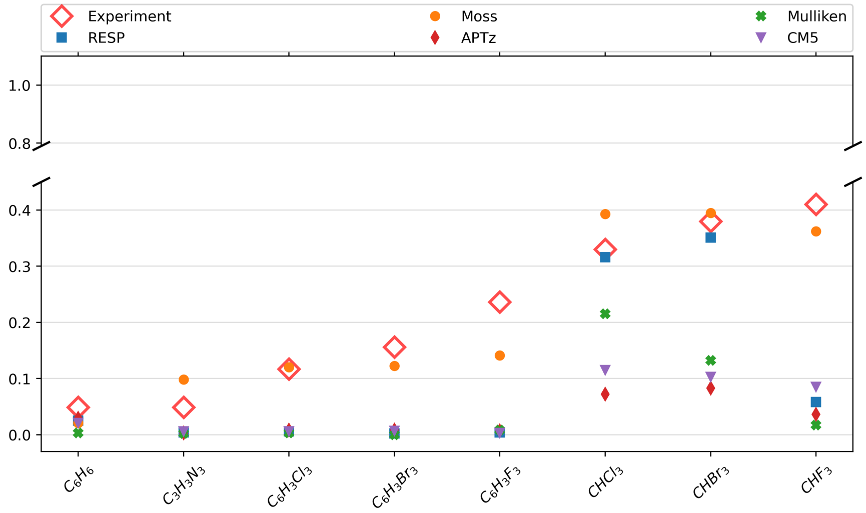

Figure S1: Vibrational g-factors calculated by the method of Moss and Perry as described in the main article and rescaled by a factor of 0.404.

S2 GFN2-xTB data for comparison

This sections provides a quick overview of the vibrational frequencies obtained with the GFN2-xTB method.

Table S1: Strongly IR-active pseudorotations of benzene, H_2-, Mn-, and Fe-phthalocyanines obtained from GFN2-xTB calculations: index, vibrational frequency, relative intensity.

index	ω / cm^{-1}	rel. int.
	C_6H_6	
3, 4	580	0
28, 29	3084	120
	H_2Pc	
121, 122	1319, 1339	219, 216
137, 138	1514, 1516	75, 228
155, 157	3085, 3091	253, 229
162, 166	3103, 3109	400, 205
	MnPc	
103, 104	1143	116
123, 124	1345	187
138, 139	1520	239
155, 156	3089	127
163, 164	3108	144
	FePc	
103, 104	1149	178
122, 123	1345	207
138, 140	1525	247
155, 156	3088	127
163, 164	3108	122

Table S2: Strongly IR-active pseudorotations of Co-, Ni-, and Cu-phtalocyanine obtained from GFN2-xTB calculations: index, vibrational frequency, relative intensity.

index	ω / cm^{-1}	rel. int.
	CoPc	
103, 104	1149	130
122, 123	1348	154
138, 139	1534	231
155, 156	3089	269
163, 164	3108	283
	NiPc	
103, 104	1149	135
122, 123	1347	170
138, 139	1524	178
155, 156	3089	226
163, 164	3109	200
	CuPc	
102, 103	1156	107
123, 124	1345	188
139, 140	1515	163
155, 156	3088	300
163, 164	3107	262

S3 IR spectra details

In this section we present and compare calculated spectra. Spectra calculated with Q-Chem employ the B97D GGA functional and the def2-SVP basis set. Spectra calculated using the GFN2-xTB approach are compared to the DFT results. Also, a comparison of the CuPc and H_2Pc DFT calculations to experiment[8–10] is provided.

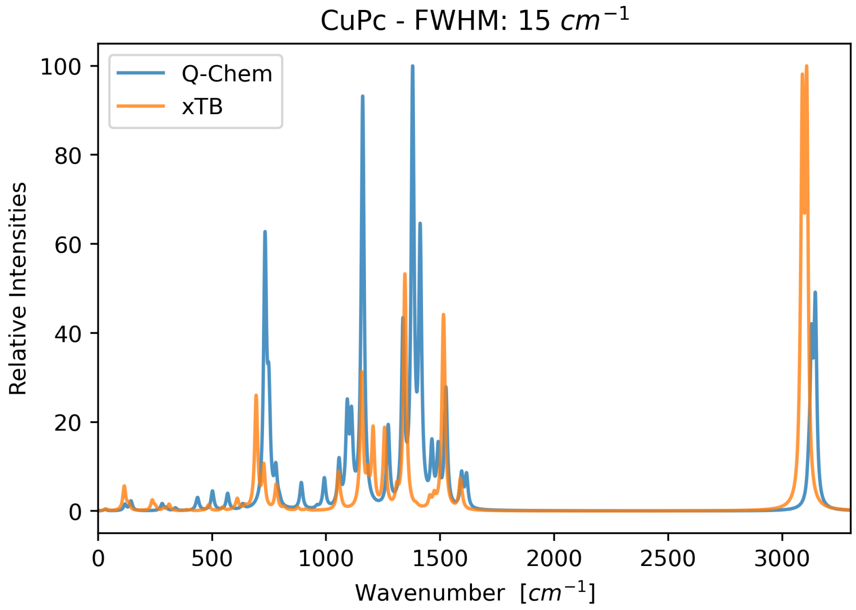

Figure S2: IR adsorption spectra of CuPc obtained from GFN-xTB and Q-Chem calculations (see main article). Vibrations above 3000 cm^{-1} correspond to motions of H atoms; the larger peaks around 1000 cm^{-1} are mostly planar motions of N atoms. A Lorenz line shape function with FWHM of 10 cm^{-1} was applied.

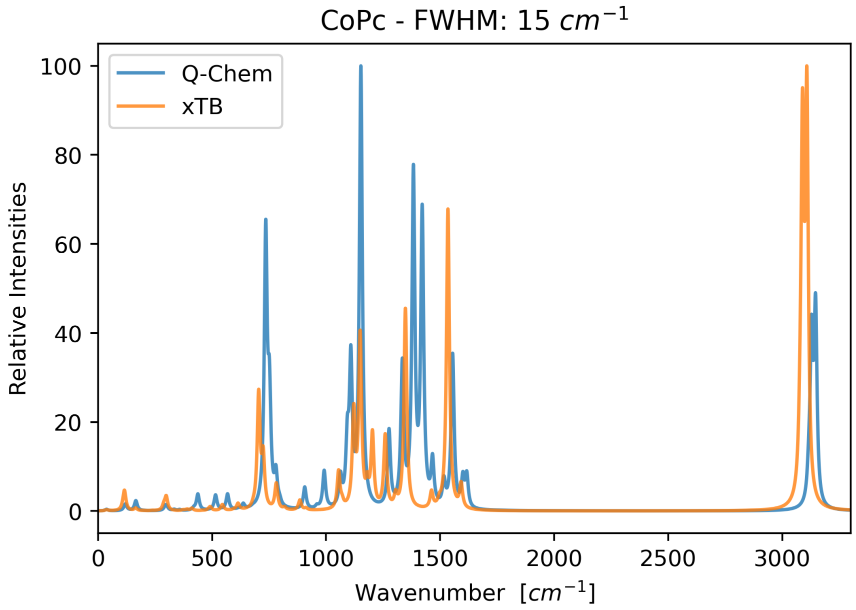

Figure S3: IR adsorption spectra of CoPc obtained from GFN-xTB and Q-Chem calculations (see main article). Vibrations above 3000 cm^{-1} correspond to motions of H atoms; the larger peaks around 1000 cm^{-1} are mostly planar motions of N atoms. A Lorenz line shape function with FWHM of 10 cm^{-1} was applied.

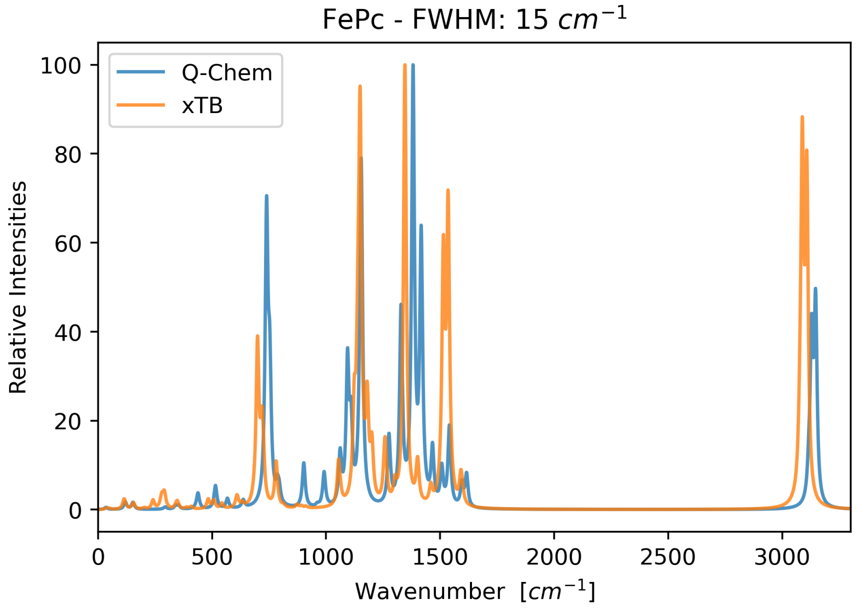

Figure S4: IR adsorption spectra of FePc obtained from GFN-xTB and Q-Chem calculations (see main article). Vibrations above 3000 cm^{-1} correspond to motions of H atoms; the larger peaks around 1000 cm^{-1} are mostly planar motions of N atoms. A Lorenz line shape function with FWHM of 10 cm^{-1} was applied.

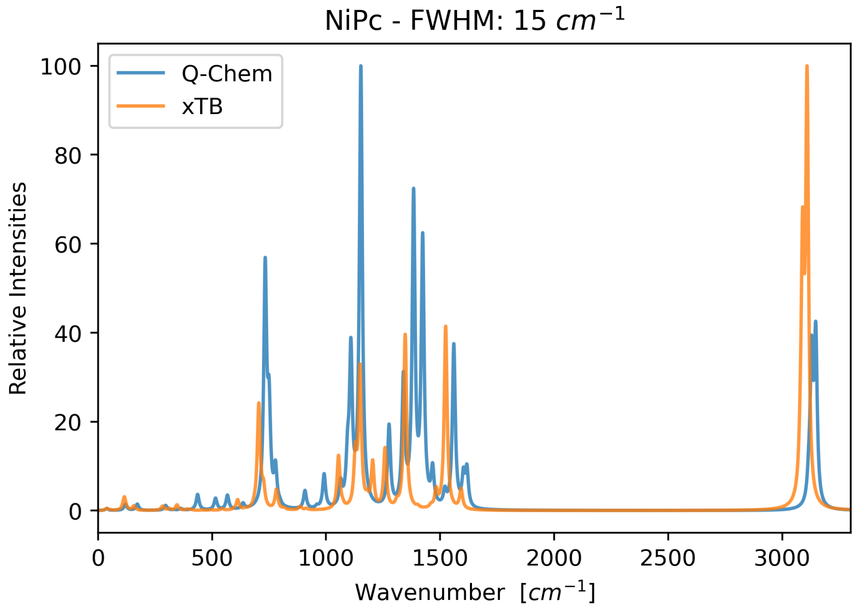

Figure S5: IR adsorption spectra of NiPc obtained from GFN-xTB and Q-Chem calculations (see main article). Vibrations above 3000 cm^{-1} correspond to motions of H atoms; the larger peaks around 1000 cm^{-1} are mostly planar motions of N atoms. A Lorenz line shape function with FWHM of 10 cm^{-1} was applied.

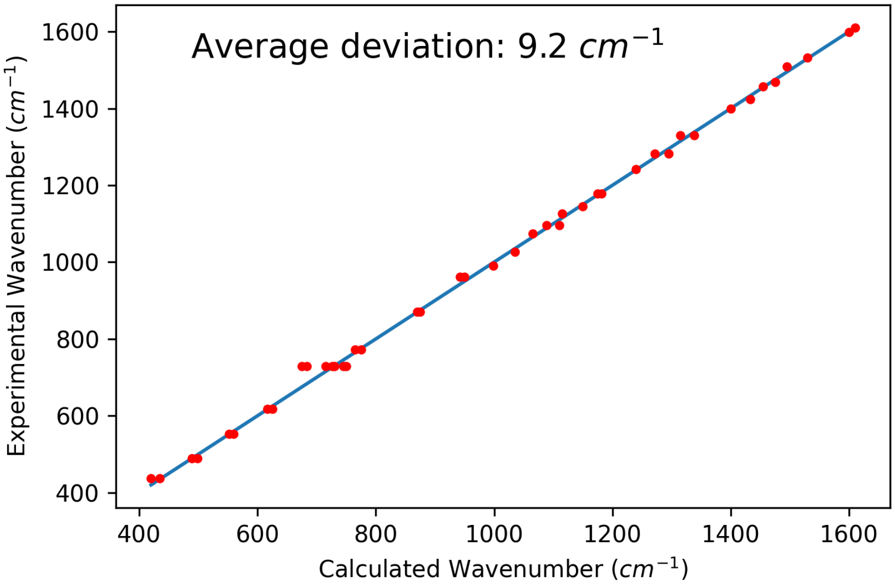

Figure S6: DFT-calculated vs experimental wavenumber for H_2Pc using a DFT calculation. A scale factor of 0.998 was applied to the calculated spectrum.

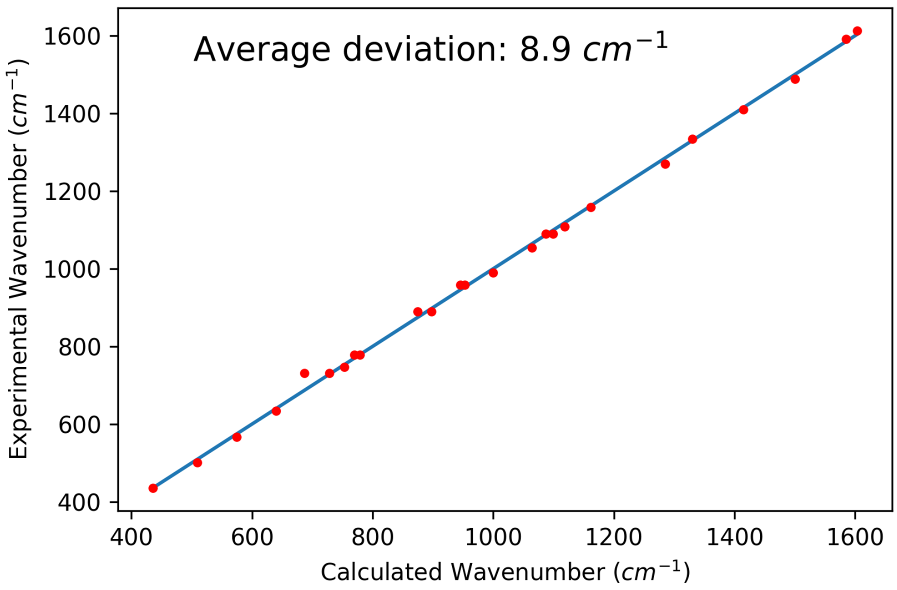

Figure S7: DFT-calculated vs experimental wavenumber for CuPc using a DFT calculation. A scale factor of 0.998 was applied to the calculated spectrum.

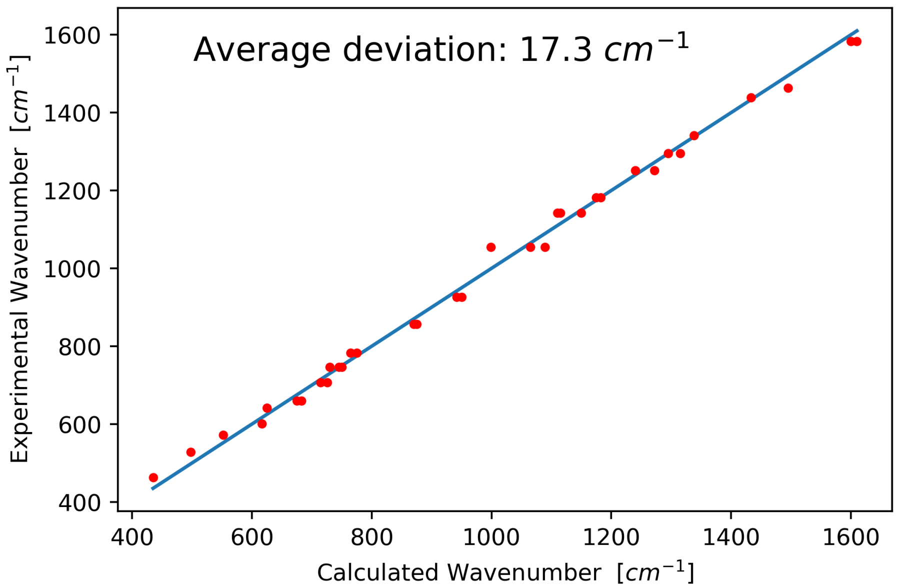

Figure S8: xTB-calculated vs experimental wavenumber for H_2Pc using a xTB calculation.

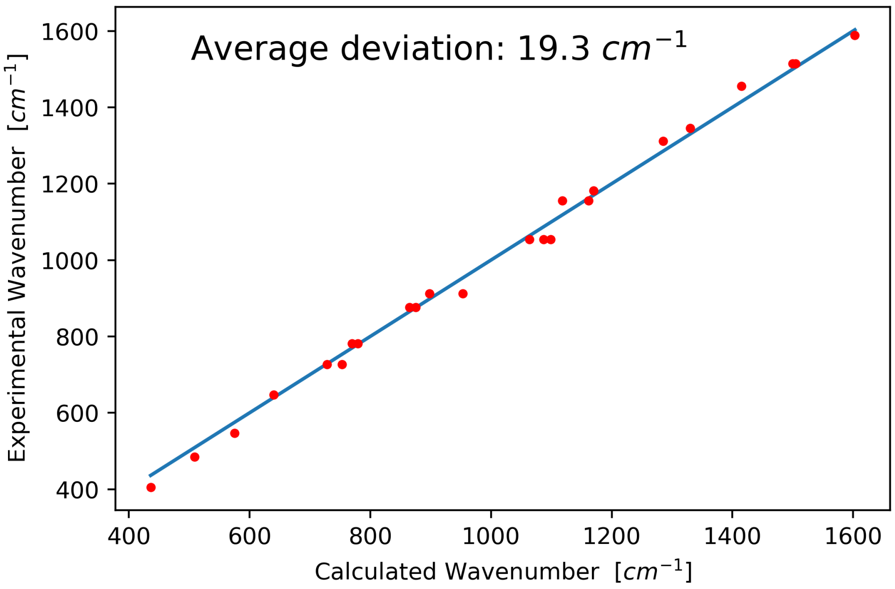

Figure S9: Calculated vs experimental wavenumber for CuPc using a xTB calculation.

S4 Electron density plots

This section contains electron density plots as they occur during pseudorotation, calculated via a DFT-approach in Q-Chem with a b97d GGA functional and def2-SVP basis set. All plots assumed a vibrational amplitude according to the standard deviation of the first excited state of the corresponding harmonic oscillator. The eigenvectors are not plotted to scale, but are enlarged.

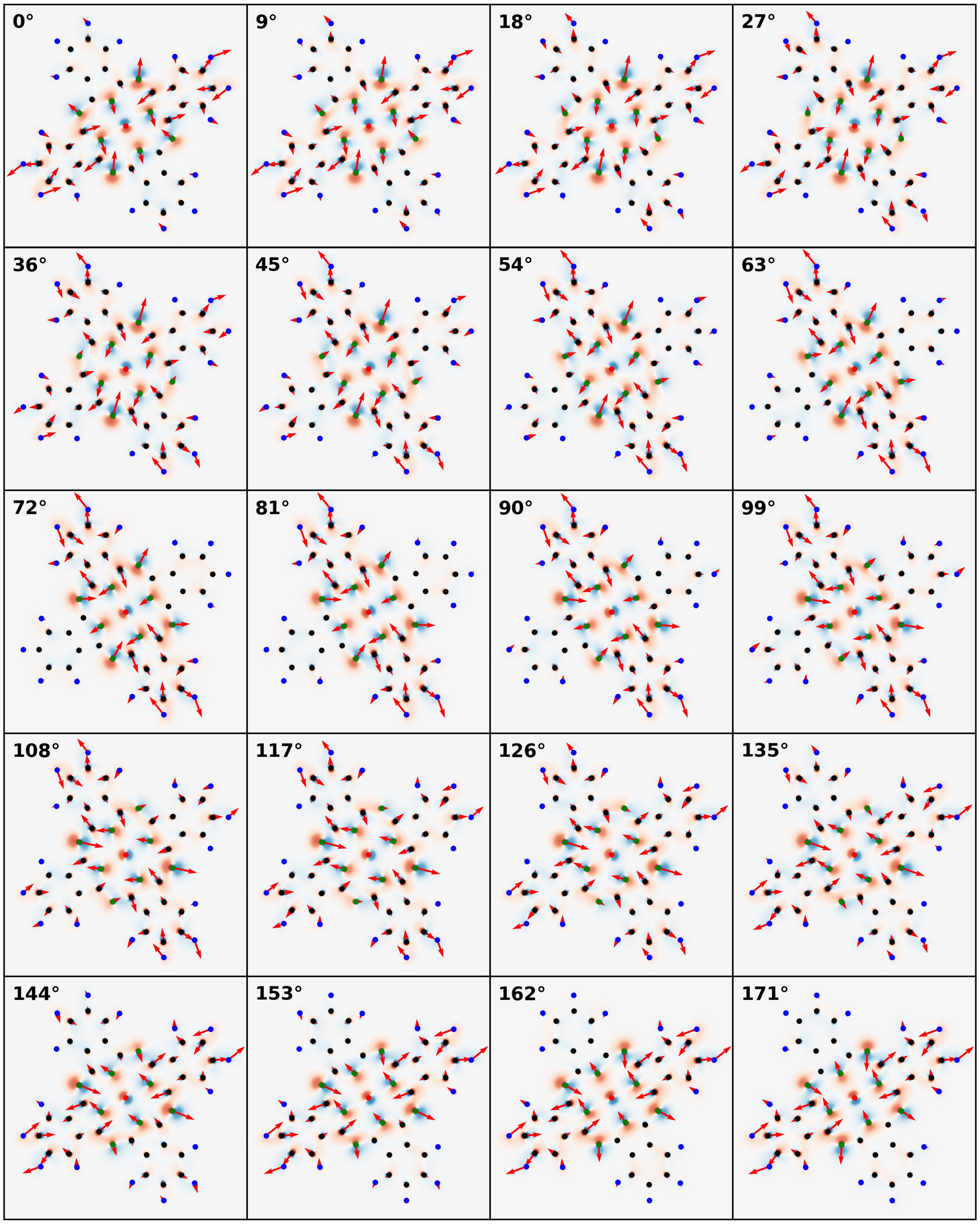

Figure S10: Change of electron density during vibration for the 891 cm^{-1} mode of CuPc. The plots are made in 9 degree steps in terms of the phase angle ωt. The electron density has been integrated along the z-direction and is plotted on a logarithmic scale. Excess and lack of electron density is denoted as blue or red. Distances in Bohr. Red arrows indicate the eigenvectors.

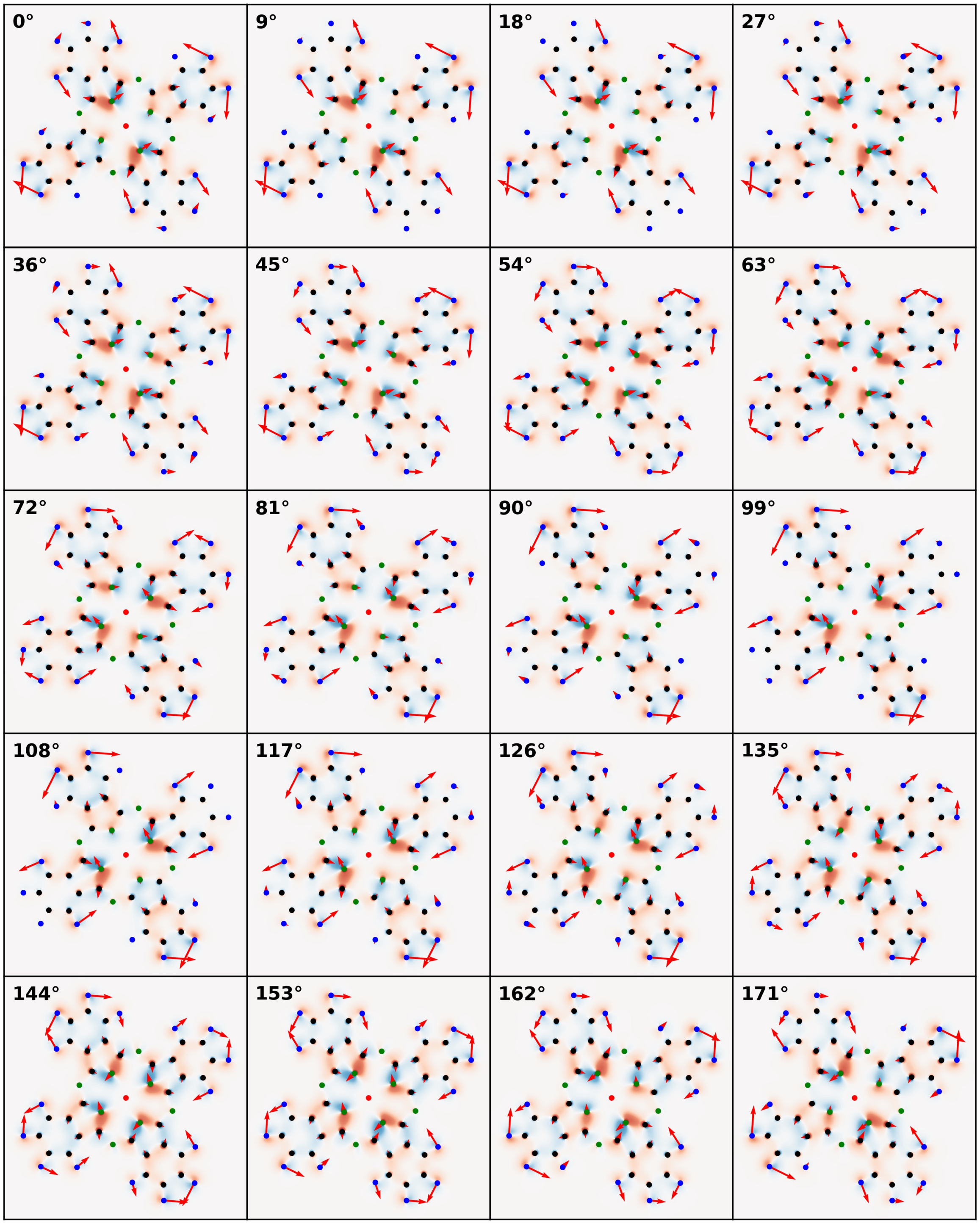

Figure S11: Change of electron density during vibration for the 1160 cm^{-1} mode of CuPc. The plots are made in 9 degree steps in terms of the phase angle ωt. The electron density has been integrated along the z-direction and is plotted on a logarithmic scale. Excess and lack of electron density is denoted as blue or red. Distances in Bohr. Red arrows indicate the eigenvectors.

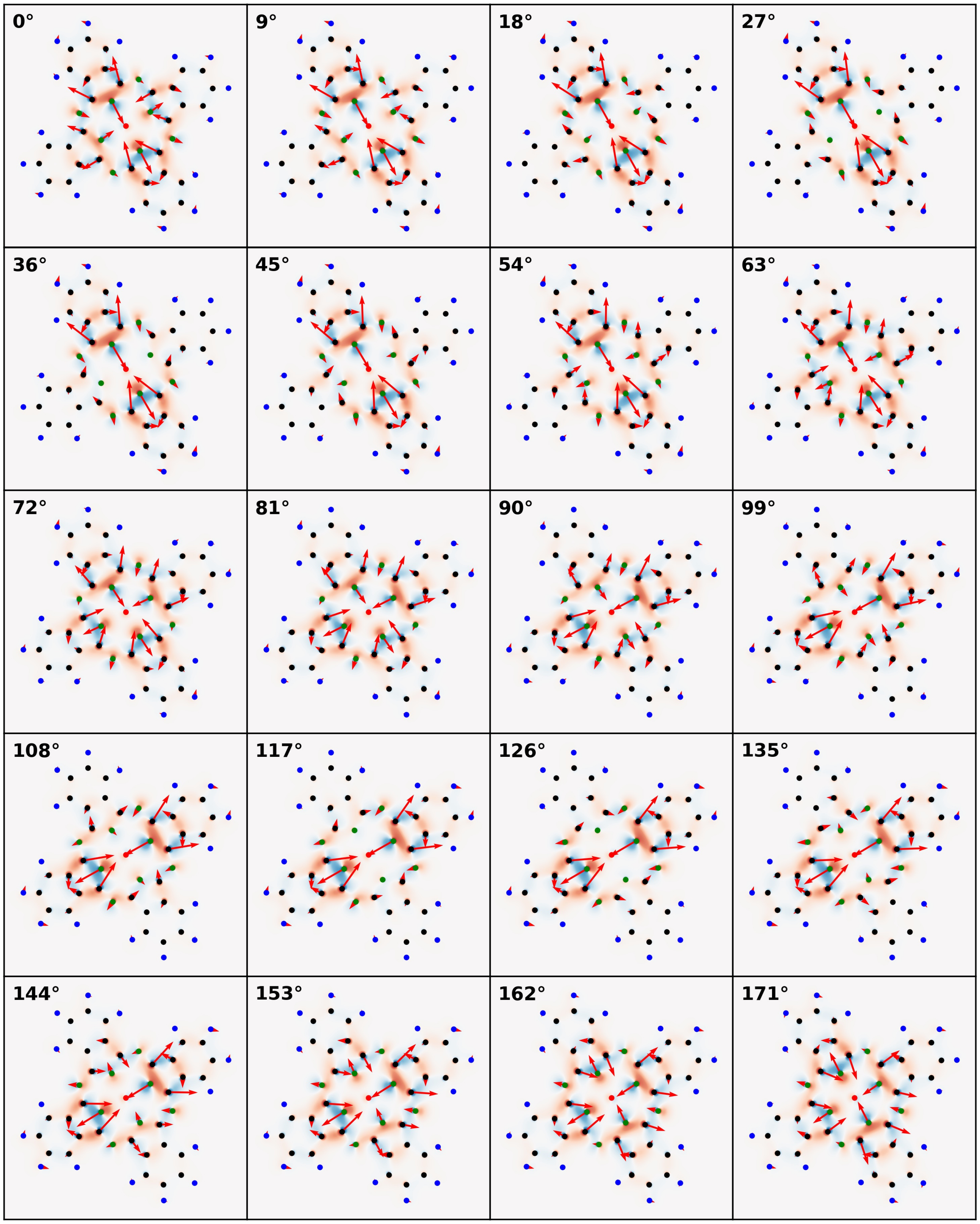

Figure S12: Change of electron density during vibration for the 1336 cm^{-1} mode of CuPc. The plots are made in 9 degree steps in terms of the phase angle ωt. The electron density has been integrated along the z-direction and is plotted on a logarithmic scale. Excess and lack of electron density is denoted as blue or red. Distances in Bohr. Red arrows indicate the eigenvectors.

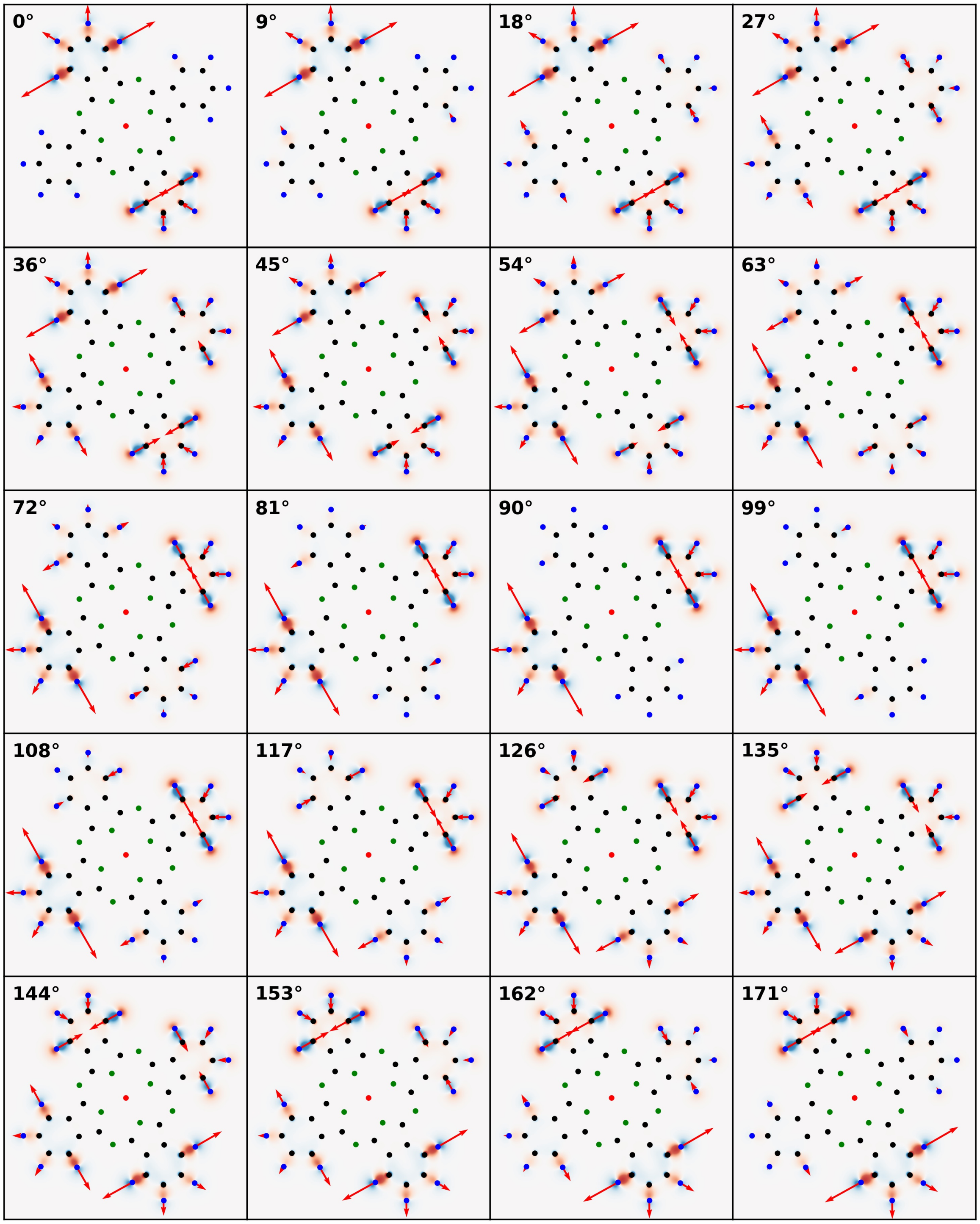

Figure S13: Change of electron density during vibration for the 3144 cm^{-1} mode of CuPc. The plots are made in 9 degree steps in terms of the phase angle ωt. The electron density has been integrated along the z-direction and is plotted on a logarithmic scale. Excess and lack of electron density is denoted as blue or red. Distances in Bohr. Red arrows indicate the eigenvectors.

S5 Electric dipole moment

S5.1 H_2Pc dipole scans

Pseudorotational excitation translates into a rotating electric dipole, as can be seen from Figure S14 below, featuring a phase difference of 90° between its two Cartesian components in the molecular plane. Note the slight deviation in amplitude in case of H_2Pc due to the reduced symmetry of the molecular system.

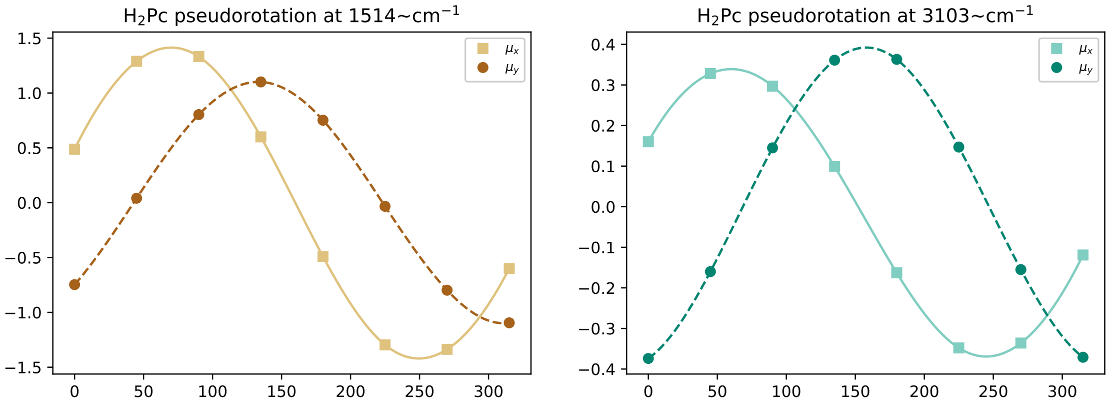

Figure S14: Dipole scans for H_2Pc...Scan over the electric dipole moment of H_2Pc as a function of the phase angle $\phi = \omega t$, corresponding to vibrational excitations at 1526 and 3144 cm^{-1}, calculated with DFT ansatz.

S5.2 Rotating dipoles interpreted via the center of charge

The following Figures show how to imagine the rotating electric dipole as arising from the change in the center of charge. As the electron density and nuclei change during vibration, also the positive and negative centers of charge change. The distance from the positive to the negative center of charge gives the direction of the electric dipole moment.

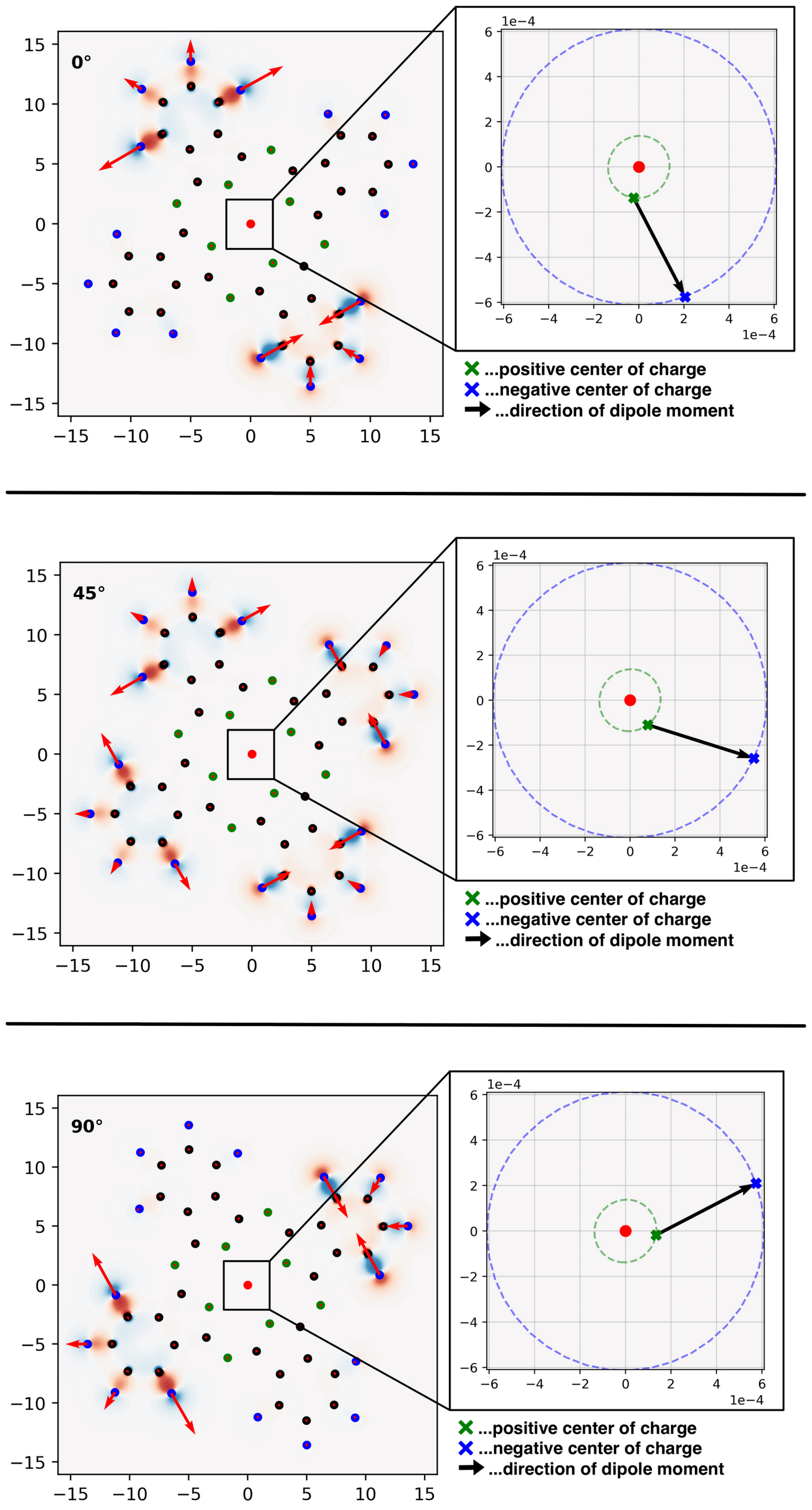

Figure S15: Change of electron density during vibration for the 3144 cm^{-1} mode of CuPc for different phase angles ωt (left). Positive and negative centers of charge and direction of the dipole moment (right).

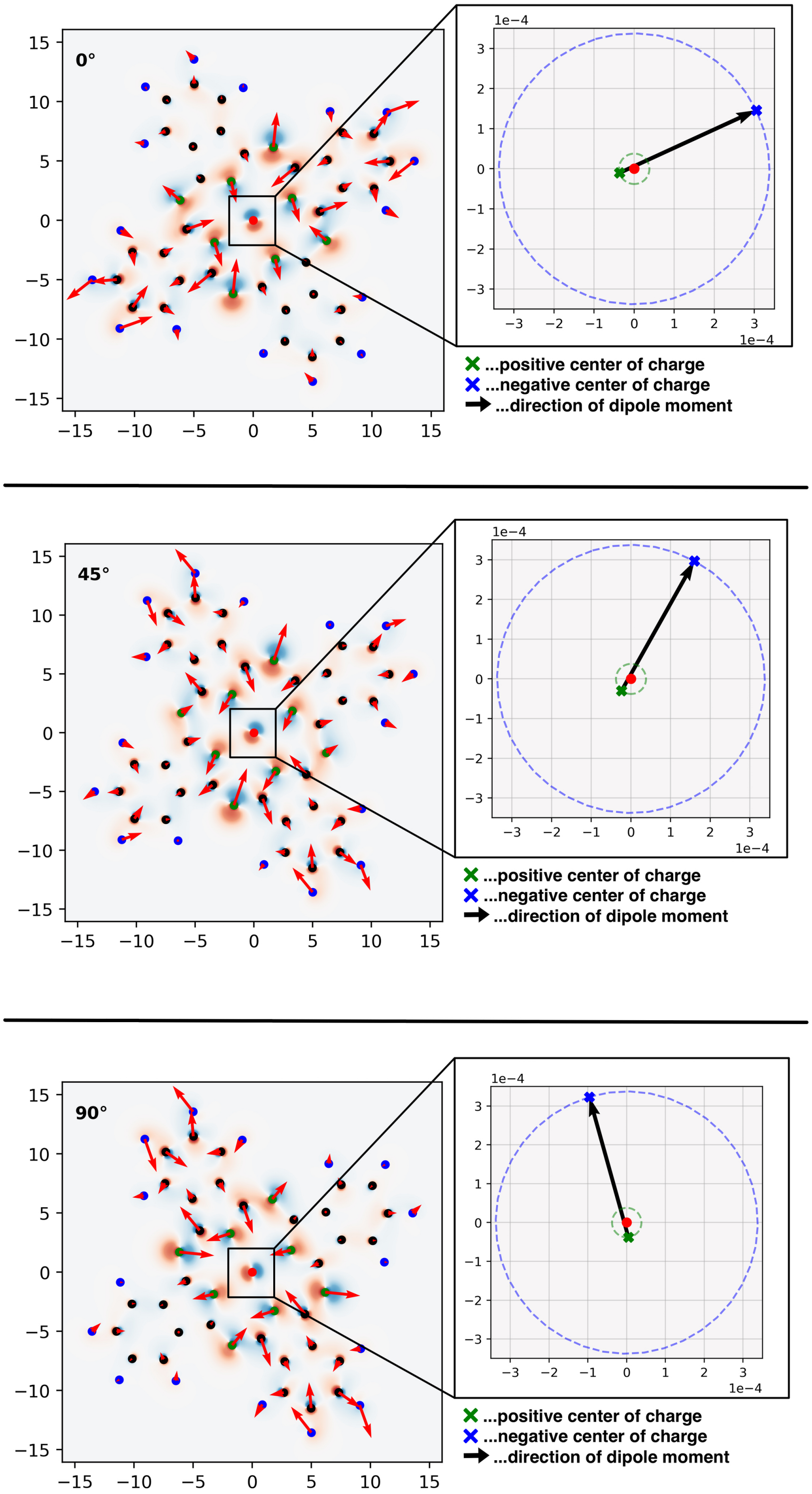

Figure S16: Change of electron density during vibration for the 891 cm^{-1} mode of CuPc for different phase angles ωt (left). Positive and negative centers of charge and direction of the dipole moment (right).

S5.3 Rotating dipoles calculated from partial charges

Figure S17 illustrates how partial charges can reproduce the electric dipole moment. In the left figure, the partial charges were calculated in the equilibrium position of the atoms (i.e. no displacement due to vibration was assumed). Then, the atomic sites where the partial charges sit were displaced from their equilibrium position according to the eigenvectors of vibration. As expected, these equilibrium partial charges do not reproduce the electric dipole moment at all. Only the APT charge, which is independent of the displacement, does reproduce the electric dipole moment.

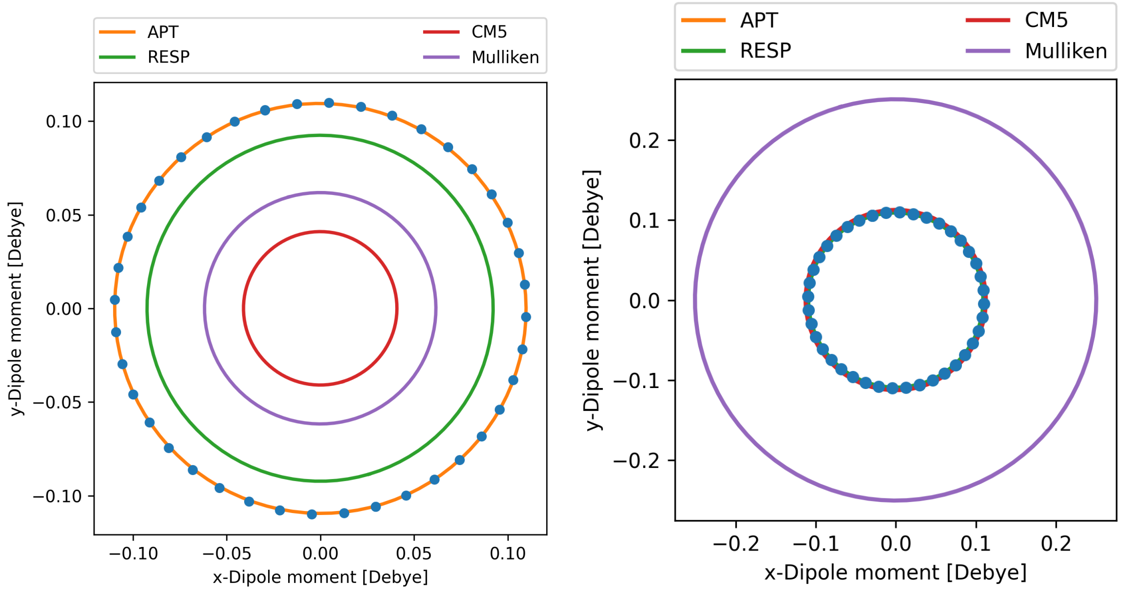

Figure S17: Electric dipole moment calculated via the use of different partial charge models for the 891 cm^{-1} mode. Blue points denote the values calculated directly by Q-Chem via DFT at different phase angles $\phi = \omega t$. Left: Partial charges were calculated for the undisturbed equilibrium geometry of the molecule. Right: Partial charges were calculated assuming a distorted molecule according to the vibrational eigenvectors. In the right plot all charges, except Mulliken partial charges, reproduce the calculated dipole moment from Q-Chem and therefore overlap.

Re-evaluating, however, the partial charges at the displaced atomic sites at every phase angle ωt leads to much better results. In the right figure, the dipole moment produced by these re-evaluated partial charges are plotted. Indeed, all but Mulliken charges reproduce the electric dipole moment within a few percentage points. Nevertheless, it has to be noted

that CM5 charges use an semi-empirical method for calculating partial charges. This semi-empirical method does not necessarily conserve charge neutrality and therefore the dipole moment. On the other hand, RESP charges are often calculated under the constraint of reproducing the electric dipole moment. Q-Chem, however, does not do this and small deviations from the exact dipole moment are expected and observed.

S6 Optimized Geometries

This section provides the optimized geometries of all the neutral phathalocyanines treated in the main article (H_2Pc, FePc, CoPc, NiPc, CuPc). These structures were calculated with an DFT approch using a b97d GGA functional and def2-SVP basis in Q-Chem The structures are given in Cartesian coordinates in units of Ångstrom. The formatting supports direct copy and paste from the pdf document.

S6.1 H_2Pc

```
58


C -0.70681 -4.19123 0.00000
C -1.43500 -5.38904 0.00000
C -0.70736 -6.59405 0.00000
C 0.70736 -6.59405 0.00000
C 1.43500 -5.38904 0.00000
C 0.70681 -4.19123 0.00000
H -1.24278 -7.55224 0.00000
H 1.24278 -7.55224 0.00000
H 2.53157 -5.37665 0.00000
H -2.53157 -5.37665 0.00000
C 1.10249 -2.77246 0.00000
N -0.00000 -1.96175 0.00000
C -1.10249 -2.77246 0.00000
N -2.39045 -2.40438 0.00000
C -6.60236 -0.70950 0.00000
C -6.60236 0.70950 0.00000
N -2.04175 0.00000 0.00000
```

```
C -5.40309 -1.43640 0.00000
C -5.40309 1.43640 0.00000
C -4.19858 -0.71157 0.00000
C -4.19858 0.71157 0.00000
H -7.56097 -1.24364 0.00000
H -7.56097 1.24364 0.00000
C -2.80753 -1.15161 0.00000
C -2.80753 1.15161 0.00000
H -5.38931 -2.53256 0.00000
H -5.38931 2.53256 0.00000
N -2.39045 2.40438 0.00000
C -0.70736 6.59405 0.00000
N 2.04175 0.00000 0.00000
C 0.70736 6.59405 0.00000
N -0.00000 1.96175 0.00000
C -1.43500 5.38904 0.00000
C 1.43500 5.38904 0.00000
C -0.70681 4.19123 0.00000
C 0.70681 4.19123 0.00000
H -1.24278 7.55224 0.00000
H 1.24278 7.55224 0.00000
C -1.10249 2.77246 0.00000
C 1.10249 2.77246 0.00000
H -2.53157 5.37665 0.00000
C 2.80753 1.15161 0.00000
H 2.53157 5.37665 0.00000
C 2.80753 -1.15161 0.00000
C 4.19858 0.71157 0.00000
C 4.19858 -0.71157 0.00000
```

```
C 5.40309 1.43640 0.00000
N 2.39045 2.40438 0.00000
C 5.40309 -1.43640 0.00000
N 2.39045 -2.40438 0.00000
C 6.60236 0.70950 0.00000
C 6.60236 -0.70950 0.00000
H 5.38931 2.53256 0.00000
H 5.38931 -2.53256 0.00000
H 7.56097 1.24364 0.00000
H 7.56097 -1.24364 0.00000
H 1.02048 0.00000 0.00000
H -1.02048 0.00000 0.00000
```

S6.2 FePc

```
57


C 0.70746 4.16643 -0.00000
C 1.43676 5.36721 0.00000
C 0.70922 6.56789 0.00000
C -0.70922 6.56789 0.00000
C -1.43676 5.36721 -0.00000
C -0.70746 4.16643 -0.00000
H 1.24332 7.52667 0.00000
H -1.24332 7.52667 0.00000
H -2.53299 5.35289 -0.00000
H 2.53299 5.35289 0.00000
C -1.12030 2.76668 -0.00000
N -0.00000 1.95216 -0.00000
C 1.12030 2.76668 -0.00000
```

```
N 2.39139 2.39139 -0.00000
C 6.56789 0.70922 -0.00000
C 6.56789 -0.70922 0.00000
N 1.95216 0.00000 0.00000
C 5.36721 1.43676 -0.00000
C 5.36721 -1.43676 0.00000
C 4.16643 0.70746 -0.00000
C 4.16643 -0.70746 0.00000
H 7.52667 1.24332 -0.00000
H 7.52667 -1.24332 0.00000
C 2.76668 1.12030 -0.00000
C 2.76668 -1.12030 0.00000
H 5.35289 2.53299 -0.00000
H 5.35289 -2.53299 0.00000
N 2.39139 -2.39139 0.00000
C 0.70922 -6.56789 -0.00000
N -1.95216 0.00000 -0.00000
C -0.70922 -6.56789 -0.00000
N -0.00000 -1.95216 0.00000
C 1.43676 -5.36721 0.00000
C -1.43676 -5.36721 -0.00000
C 0.70746 -4.16643 0.00000
C -0.70746 -4.16643 0.00000
H 1.24332 -7.52667 -0.00000
H -1.24332 -7.52667 -0.00000
C 1.12030 -2.76668 0.00000
C -1.12030 -2.76668 0.00000
H 2.53299 -5.35289 0.00000
C -2.76668 -1.12030 0.00000
```

```
H -2.53299 -5.35289 -0.00000
C -2.76668 1.12030 -0.00000
C -4.16642 -0.70746 0.00000
C -4.16642 0.70746 -0.00000
C -5.36720 -1.43676 0.00000
N -2.39139 -2.39139 0.00000
C -5.36720 1.43676 -0.00000
N -2.39139 2.39139 -0.00000
C -6.56789 -0.70922 0.00000
C -6.56789 0.70922 -0.00000
H -5.35289 -2.53299 0.00000
H -5.35289 2.53299 -0.00000
H -7.52667 -1.24332 0.00000
H -7.52667 1.24332 -0.00000
Fe -0.00000 0.00000 0.00000
```

S6.3 CoPc

```
57

C 0.70747 4.15212 0.00000
C 1.43803 5.35429 0.00000
C 0.71048 6.55255 0.00000
C -0.71048 6.55255 0.00000
C -1.43803 5.35429 0.00000
C -0.70747 4.15212 0.00000
H 1.24364 7.51185 0.00000
H -1.24364 7.51185 0.00000
H -2.53414 5.33981 0.00000
H 2.53414 5.33981 0.00000
```

```
C -1.11957 2.75776 0.00000
N 0.00000 1.93766 0.00000
C 1.11957 2.75776 0.00000
N 2.38981 2.38693 0.00000
C 6.55555 0.70832 0.00000
C 6.55555 -0.70832 0.00000
N 1.92852 0.00000 0.00000
C 5.35293 1.43595 0.00000
C 5.35293 -1.43595 0.00000
C 4.15466 0.70516 0.00000
C 4.15466 -0.70516 0.00000
H 7.51378 1.24339 0.00000
H 7.51378 -1.24339 0.00000
C 2.75333 1.11713 0.00000
C 2.75333 -1.11713 0.00000
H 5.33796 2.53215 0.00000
H 5.33796 -2.53215 0.00000
N 2.38981 -2.38693 0.00000
C 0.71048 -6.55255 0.00000
N -1.92852 0.00000 0.00000
C -0.71048 -6.55255 0.00000
N 0.00000 -1.93766 0.00000
C 1.43803 -5.35429 0.00000
C -1.43803 -5.35429 0.00000
C 0.70747 -4.15212 0.00000
C -0.70747 -4.15212 0.00000
H 1.24364 -7.51185 0.00000
H -1.24364 -7.51185 0.00000
C 1.11957 -2.75776 0.00000
```

```
C -1.11957 -2.75776 0.00000
H 2.53414 -5.33981 0.00000
C -2.75333 -1.11713 0.00000
H -2.53414 -5.33981 0.00000
C -2.75333 1.11713 0.00000
C -4.15466 -0.70516 0.00000
C -4.15466 0.70516 0.00000
C -5.35293 -1.43595 0.00000
N -2.38981 -2.38693 0.00000
C -5.35293 1.43595 0.00000
N -2.38981 2.38693 0.00000
C -6.55555 -0.70832 0.00000
C -6.55555 0.70832 0.00000
H -5.33796 -2.53215 0.00000
H -5.33796 2.53215 0.00000
H -7.51378 -1.24339 0.00000
H -7.51378 1.24339 0.00000
Co 0.00000 0.00000 0.00000
```

S6.4 NiPc

```
57

C 0.70517 4.14776 0.00000
C 1.43690 5.34678 0.00000
C 0.70910 6.54754 0.00000
C -0.70910 6.54754 0.00000
C -1.43690 5.34678 0.00000
C -0.70517 4.14776 0.00000
H 1.24340 7.50619 0.00000
```

```
H -1.24340 7.50619 0.00000
H -2.53305 5.33175 0.00000
H 2.53305 5.33175 0.00000
C -1.11602 2.74864 0.00000
N -0.00000 1.92621 0.00000
C 1.11602 2.74864 0.00000
N 2.38574 2.38574 0.00000
C 6.54754 0.70910 0.00000
C 6.54754 -0.70910 0.00000
N 1.92621 0.00000 0.00000
C 5.34678 1.43690 0.00000
C 5.34678 -1.43690 0.00000
C 4.14776 0.70517 0.00000
C 4.14776 -0.70517 0.00000
H 7.50619 1.24340 0.00000
H 7.50619 -1.24340 0.00000
C 2.74864 1.11602 0.00000
C 2.74865 -1.11602 0.00000
H 5.33175 2.53305 0.00000
H 5.33175 -2.53305 0.00000
N 2.38574 -2.38574 0.00000
C 0.70910 -6.54754 0.00000
N -1.92621 0.00000 0.00000
C -0.70910 -6.54754 0.00000
N -0.00000 -1.92621 0.00000
C 1.43690 -5.34678 0.00000
C -1.43690 -5.34678 0.00000
C 0.70517 -4.14776 0.00000
C -0.70517 -4.14776 0.00000
```

```
H 1.24340 -7.50619 0.00000
H -1.24340 -7.50619 0.00000
C 1.11602 -2.74864 0.00000
C -1.11602 -2.74864 0.00000
H 2.53305 -5.33175 0.00000
C -2.74864 -1.11602 0.00000
H -2.53305 -5.33175 0.00000
C -2.74864 1.11602 0.00000
C -4.14776 -0.70517 0.00000
C -4.14776 0.70517 0.00000
C -5.34678 -1.43690 0.00000
N -2.38574 -2.38574 0.00000
C -5.34678 1.43690 0.00000
N -2.38574 2.38574 0.00000
C -6.54754 -0.70910 0.00000
C -6.54754 0.70910 0.00000
H -5.33175 -2.53305 0.00000
H -5.33175 2.53305 0.00000
H -7.50619 -1.24340 0.00000
H -7.50619 1.24340 0.00000
Ni -0.00000 0.00000 0.00000
```

S6.5 CuPc

```
57

C 0.70853 4.18888 0.00000
C 1.43593 5.38899 0.00000
C 0.70867 6.59106 0.00000
C -0.70867 6.59106 -0.00000
```

```
C -1.43593 5.38899 -0.00000
C -0.70853 4.18888 -0.00000
H 1.24389 7.54924 0.00000
H -1.24389 7.54924 -0.00000
H -2.53205 5.37306 -0.00000
H 2.53205 5.37306 0.00000
C -1.12471 2.78402 -0.00000
N 0.00000 1.98843 0.00000
C 1.12471 2.78402 0.00000
N 2.39540 2.39540 0.00000
C 6.59106 0.70867 0.00000
C 6.59106 -0.70867 0.00000
N 1.98843 -0.00000 0.00000
C 5.38899 1.43593 0.00000
C 5.38899 -1.43593 0.00000
C 4.18888 0.70853 0.00000
C 4.18888 -0.70853 0.00000
H 7.54924 1.24389 0.00000
H 7.54924 -1.24389 0.00000
C 2.78402 1.12471 0.00000
C 2.78402 -1.12471 0.00000
H 5.37306 2.53205 0.00000
H 5.37306 -2.53205 0.00000
N 2.39540 -2.39540 0.00000
C 0.70867 -6.59106 0.00000
N -1.98843 0.00000 -0.00000
C -0.70867 -6.59106 -0.00000
N -0.00000 -1.98843 -0.00000
C 1.43593 -5.38899 0.00000
```

```
C -1.43593 -5.38899 -0.00000
C 0.70853 -4.18888 0.00000
C -0.70853 -4.18888 -0.00000
H 1.24389 -7.54924 0.00000
H -1.24389 -7.54924 -0.00000
C 1.12471 -2.78402 0.00000
C -1.12471 -2.78402 -0.00000
H 2.53205 -5.37306 0.00000
C -2.78402 -1.12471 -0.00000
H -2.53205 -5.37306 -0.00000
C -2.78402 1.12471 -0.00000
C -4.18888 -0.70853 -0.00000
C -4.18888 0.70853 -0.00000
C -5.38899 -1.43593 -0.00000
N -2.39540 -2.39540 -0.00000
C -5.38899 1.43593 -0.00000
N -2.39540 2.39540 -0.00000
C -6.59106 -0.70867 -0.00000
C -6.59106 0.70867 -0.00000
H -5.37306 -2.53205 -0.00000
H -5.37306 2.53205 -0.00000
H -7.54924 -1.24389 -0.00000
H -7.54924 1.24389 -0.00000
Cu 0.00000 0.00000 0.00000
```

S7 Vibrational Eigenmodes

This section contains excerpts of the CoPc output files obtained with the Q-Chem program package as used for the calculation of vibrational g-factors. It provides the equilibrium positions, some vibrational frequencies and their corresponding eigenmodes.

S7.1 891 cm^{-1}

Table S3: Eigenvectors for the 891 cm^{-1} eigenmode of CuPc. Each eigenvector is normalized to one and not mass-weighted. $\vec{a}_i$ and $\vec{b}_i$ correspond to the eigenvector components pointing into one of the two planar Cartesian coordinate directions. The positions of the atoms in space are denoted as x, y, z with units in Angstrom.

**Atom**	**x**	**y**	**z**	$\vec{a}_x$	$\vec{a}_y$	$\vec{a}_z$	$\vec{b}_x$	$\vec{b}_y$	$\vec{b}_z$
C	0.708	4.188	0.000	0.001	0.085	0.000	-0.003	-0.002	-0.000
C	1.436	5.389	0.000	0.088	-0.041	-0.000	0.040	0.015	0.000
C	0.708	6.591	0.000	-0.081	-0.173	-0.000	0.009	0.007	0.000
C	-0.708	6.591	0.000	-0.080	0.172	0.000	-0.015	0.022	-0.000
C	-1.436	5.389	0.000	0.092	0.039	0.000	-0.032	0.018	-0.000
C	-0.708	4.188	0.000	0.000	-0.085	-0.000	0.003	-0.009	0.000
H	1.243	7.549	0.000	0.041	-0.243	0.000	-0.009	0.018	-0.000
H	-1.243	7.549	0.000	0.040	0.241	-0.000	0.012	0.039	0.000
H	-2.532	5.376	0.000	0.090	-0.047	-0.000	-0.034	0.027	0.000
H	2.532	5.376	0.000	0.086	0.044	0.000	0.041	0.031	-0.000
C	-1.124	2.784	0.000	0.039	-0.216	0.000	-0.001	-0.027	-0.000
N	0.000	1.988	0.000	0.181	0.007	-0.000	0.008	-0.153	-0.000
C	1.124	2.784	0.000	0.039	0.217	0.000	0.005	-0.008	0.000
N	2.395	2.395	0.000	-0.188	-0.111	-0.000	0.096	0.179	-0.000
C	6.591	0.708	0.000	-0.022	-0.015	-0.000	0.172	0.080	-0.000
C	6.591	-0.708	0.000	-0.007	0.009	0.000	-0.173	0.081	0.000

N	1.988	0.000	0.000	0.153	0.008	0.000	0.007	-0.181	-0.000
C	5.389	1.436	0.000	-0.018	-0.032	-0.000	0.039	-0.092	-0.000
C	5.389	-1.436	0.000	-0.015	0.040	0.000	-0.041	-0.088	0.000
C	4.188	0.708	0.000	0.009	0.003	0.000	-0.085	-0.000	0.000
C	4.188	-0.708	0.000	0.002	-0.003	-0.000	0.085	-0.001	-0.000
H	7.549	1.243	0.000	-0.039	0.012	0.000	0.241	-0.040	0.000
H	7.549	-1.243	0.000	-0.018	-0.009	-0.000	-0.243	-0.041	-0.000
C	2.784	1.124	0.000	0.027	-0.001	-0.000	-0.216	-0.039	-0.000
C	2.784	-1.124	0.000	0.008	0.005	0.000	0.217	-0.039	0.000
H	5.376	2.532	0.000	-0.027	-0.034	0.000	-0.047	-0.090	0.000
H	5.376	-2.532	0.000	-0.031	0.041	-0.000	0.044	-0.086	-0.000
N	2.395	-2.395	0.000	-0.179	0.096	-0.000	-0.111	0.188	-0.000
C	0.708	-6.591	0.000	-0.080	0.172	0.000	-0.015	0.022	0.000
N	-1.988	-0.000	0.000	0.153	0.008	0.000	0.007	-0.181	0.000
C	-0.708	-6.591	0.000	-0.081	-0.173	-0.000	0.009	0.007	-0.000
N	-0.000	-1.988	0.000	0.181	0.007	0.000	0.008	-0.153	0.000
C	1.436	-5.389	0.000	0.092	0.039	0.000	-0.032	0.018	0.000
C	-1.436	-5.389	0.000	0.088	-0.041	-0.000	0.040	0.015	-0.000
C	0.708	-4.188	0.000	0.000	-0.085	-0.000	0.003	-0.009	-0.000
C	-0.708	-4.188	0.000	0.001	0.085	0.000	-0.003	-0.002	0.000
H	1.243	-7.549	0.000	0.040	0.241	-0.000	0.012	0.039	-0.000
H	-1.243	-7.549	0.000	0.041	-0.243	0.000	-0.009	0.018	0.000
C	1.124	-2.784	0.000	0.039	-0.216	0.000	-0.001	-0.027	0.000
C	-1.124	-2.784	0.000	0.039	0.217	-0.000	0.005	-0.008	-0.000
H	2.532	-5.376	0.000	0.090	-0.047	-0.000	-0.034	0.027	-0.000
C	-2.784	-1.124	0.000	0.027	-0.001	-0.000	-0.216	-0.039	-0.000
H	-2.532	-5.376	0.000	0.086	0.044	0.000	0.041	0.031	0.000

C	-2.784	1.124	0.000	0.008	0.005	0.000	0.217	-0.039	0.000
C	-4.188	-0.708	0.000	0.009	0.003	0.000	-0.085	-0.000	0.000
C	-4.188	0.708	0.000	0.002	-0.003	-0.000	0.085	-0.001	-0.000
C	-5.389	-1.436	0.000	-0.018	-0.032	-0.000	0.039	-0.092	-0.000
N	-2.395	-2.395	0.000	-0.188	-0.111	0.000	0.096	0.179	0.000
C	-5.389	1.436	0.000	-0.015	0.040	0.000	-0.041	-0.088	0.000
N	-2.395	2.395	0.000	-0.179	0.096	-0.000	-0.111	0.188	-0.000
C	-6.591	-0.708	0.000	-0.022	-0.015	-0.000	0.172	0.080	-0.000
C	-6.591	0.708	0.000	-0.007	0.009	0.000	-0.173	0.081	0.000
H	-5.376	-2.532	0.000	-0.027	-0.034	-0.000	-0.047	-0.090	0.000
H	-5.376	2.532	0.000	-0.031	0.041	-0.000	0.044	-0.086	-0.000
H	-7.549	-1.243	0.000	-0.039	0.012	0.000	0.241	-0.040	0.000
H	-7.549	1.243	0.000	-0.018	-0.009	-0.000	-0.243	-0.041	-0.000
Cu	0.000	0.000	0.000	-0.022	-0.001	-0.000	-0.001	0.022	-0.000

S7.2 1160 cm^{-1}

Table S4: Eigenvectors for the 1160 cm^{-1} eigenmode of CuPc. Each eigenvector is normalized to one and not mass-weighted. $\vec{a}_i$ and $\vec{b}_i$ correspond to the eigenvector components pointing into one of the two planar Cartesian coordinate directions. The positions of the atoms in space are denoted as x, y, z with units in Angstrom

**Atom**	**x**	**y**	**z**	$\vec{a}_x$	$\vec{a}_y$	$\vec{a}_z$	$\vec{b}_x$	$\vec{b}_y$	$\vec{b}_z$
C	0.708	4.188	0.00	-0.043	0.073	-0.000	-0.029	-0.027	-0.000
C	1.436	5.389	0.00	-0.011	0.014	-0.000	0.028	0.010	0.000
C	0.708	6.591	0.00	0.017	-0.035	0.000	0.005	0.008	-0.000
C	-0.708	6.591	0.00	-0.013	-0.035	-0.000	0.012	0.010	-0.000
C	-1.436	5.389	0.00	0.023	0.008	0.000	0.020	-0.015	0.000

C	-0.708	4.188	0.00	0.025	0.078	-0.000	-0.046	-0.010	-0.000
H	1.243	7.549	0.00	0.293	-0.193	-0.000	-0.056	0.043	0.000
H	-1.243	7.549	0.00	-0.285	-0.190	-0.000	0.090	0.054	0.000
H	-2.532	5.376	0.00	0.028	-0.036	-0.000	0.022	-0.280	-0.000
H	2.532	5.376	0.00	-0.014	0.101	0.000	0.033	0.264	0.000
C	-1.124	2.784	0.00	-0.053	0.012	0.000	-0.079	0.070	0.000
N	0.000	1.988	0.00	0.041	-0.047	-0.000	0.162	0.012	0.000
C	1.124	2.784	0.00	0.009	-0.022	0.000	-0.095	-0.068	-0.000
N	2.395	2.395	0.00	-0.000	-0.010	-0.000	-0.011	0.005	-0.000
C	6.591	0.708	0.00	-0.010	0.012	-0.000	-0.035	0.013	0.000
C	6.591	-0.708	0.00	-0.008	0.005	-0.000	-0.035	-0.017	0.000
N	1.988	0.000	0.00	-0.012	0.162	-0.000	-0.047	-0.041	0.000
C	5.389	1.436	0.00	0.015	0.020	0.000	0.008	-0.023	-0.000
C	5.389	-1.436	0.00	-0.010	0.028	0.000	0.014	0.011	-0.000
C	4.188	0.708	0.00	0.010	-0.046	-0.000	0.078	-0.025	0.000
C	4.188	-0.708	0.00	0.027	-0.029	-0.000	0.073	0.043	0.000
H	7.549	1.243	0.00	-0.054	0.090	0.000	-0.190	0.285	-0.000
H	7.549	-1.243	0.00	-0.043	-0.056	0.000	-0.193	-0.293	-0.000
C	2.784	1.124	0.00	-0.070	-0.079	0.000	0.012	0.053	-0.000
C	2.784	-1.124	0.00	0.068	-0.095	0.000	-0.022	-0.009	0.000
H	5.376	2.532	0.00	0.280	0.022	0.000	-0.036	-0.028	0.000
H	5.376	-2.532	0.00	-0.264	0.033	-0.000	0.101	0.014	0.000
N	2.395	-2.395	0.00	-0.005	-0.011	-0.000	-0.010	0.000	-0.000
C	0.708	-6.591	0.00	-0.013	-0.035	-0.000	0.012	0.010	0.000
N	-1.988	-0.000	0.00	-0.012	0.162	-0.000	-0.047	-0.041	0.000
C	-0.708	-6.591	0.00	0.017	-0.035	0.000	0.005	0.008	-0.000

N	-0.000	-1.988	0.00	0.041	-0.047	0.000	0.162	0.012	0.000
C	1.436	-5.389	0.00	0.023	0.008	0.000	0.020	-0.015	-0.000
C	-1.436	-5.389	0.00	-0.011	0.014	-0.000	0.028	0.010	0.000
C	0.708	-4.188	0.00	0.025	0.078	-0.000	-0.046	-0.010	0.000
C	-0.708	-4.188	0.00	-0.043	0.073	0.000	-0.029	-0.027	0.000
H	1.243	-7.549	0.00	-0.285	-0.190	0.000	0.090	0.054	-0.000
H	-1.243	-7.549	0.00	0.293	-0.193	0.000	-0.056	0.043	-0.000
C	1.124	-2.784	0.00	-0.053	0.012	0.000	-0.079	0.070	-0.000
C	-1.124	-2.784	0.00	0.009	-0.022	-0.000	-0.095	-0.068	-0.000
H	2.532	-5.376	0.00	0.028	-0.036	-0.000	0.022	-0.280	0.000
C	-2.784	-1.124	0.00	-0.070	-0.079	0.000	0.012	0.053	-0.000
H	-2.532	-5.376	0.00	-0.014	0.101	-0.000	0.033	0.264	-0.000
C	-2.784	1.124	0.00	0.068	-0.095	0.000	-0.022	-0.009	-0.000
C	-4.188	-0.708	0.00	0.010	-0.046	0.000	0.078	-0.025	0.000
C	-4.188	0.708	0.00	0.027	-0.029	-0.000	0.073	0.043	-0.000
C	-5.389	-1.436	0.00	0.015	0.020	-0.000	0.008	-0.023	-0.000
N	-2.395	-2.395	0.00	-0.000	-0.010	-0.000	-0.011	0.005	0.000
C	-5.389	1.436	0.00	-0.010	0.028	-0.000	0.014	0.011	0.000
N	-2.395	2.395	0.00	-0.005	-0.011	-0.000	-0.010	0.000	0.000
C	-6.591	-0.708	0.00	-0.010	0.012	0.000	-0.035	0.013	0.000
C	-6.591	0.708	0.00	-0.008	0.005	-0.000	-0.035	-0.017	-0.000
H	-5.376	-2.532	0.00	0.280	0.022	0.000	-0.036	-0.028	0.000
H	-5.376	2.532	0.00	-0.264	0.033	0.000	0.101	0.014	-0.000
H	-7.549	-1.243	0.00	-0.054	0.090	-0.000	-0.190	0.285	-0.000
H	-7.549	1.243	0.00	-0.043	-0.056	0.000	-0.193	-0.293	-0.000
Cu	0.000	0.000	0.00	-0.000	-0.000	0.000	-0.000	0.000	-0.000

S7.3 1336 cm^{-1}

Table S5: Eigenvectors for the 1336 cm^{-1} eigenmode of CuPc. Each eigenvector is normalized to one and not mass-weighted. $\vec{a}_i$ and $\vec{b}_i$ correspond to the eigenvector components pointing into one of the two planar Cartesian coordinate directions. The positions of the atoms in space are denoted as x, y, z with units in Angstrom

Atom	x	y	z	$\vec{a}_x$	$\vec{a}_y$	$\vec{a}_z$	$\vec{b}_x$	$\vec{b}_y$	$\vec{b}_z$
C	0.708	4.188	0.00	-0.111	0.069	-0.000	0.082	-0.044	-0.000
C	1.436	5.389	0.00	0.014	0.044	-0.000	0.005	-0.040	0.000
C	0.708	6.591	0.00	0.011	-0.030	-0.000	-0.016	0.024	-0.000
C	-0.708	6.591	0.00	-0.013	-0.016	0.000	0.015	0.035	-0.000
C	-1.436	5.389	0.00	0.008	0.028	0.000	0.012	-0.052	0.000
C	-0.708	4.188	0.00	0.053	0.026	-0.000	-0.127	-0.077	-0.000
H	1.243	7.549	0.00	0.048	-0.052	-0.000	-0.032	0.035	0.000
H	-1.243	7.549	0.00	-0.019	-0.022	-0.000	0.055	0.059	0.000
H	-2.532	5.376	0.00	0.011	-0.046	-0.000	0.011	-0.011	-0.000
H	2.532	5.376	0.00	0.014	0.022	0.000	0.008	0.042	0.000
C	-1.124	2.784	0.00	0.156	-0.159	-0.000	-0.065	0.292	0.000
N	0.000	1.988	0.00	0.018	0.211	0.000	0.014	-0.270	0.000
C	1.124	2.784	0.00	-0.025	-0.245	0.000	0.167	0.224	-0.000
N	2.395	2.395	0.00	-0.061	0.038	-0.000	-0.087	-0.099	-0.000
C	6.591	0.708	0.00	0.035	-0.015	-0.000	0.016	-0.013	0.000
C	6.591	-0.708	0.00	0.024	0.016	-0.000	0.030	0.011	0.000
N	1.988	0.000	0.00	-0.270	-0.014	-0.000	-0.211	0.018	0.000
C	5.389	1.436	0.00	-0.052	-0.012	-0.000	-0.028	0.008	-0.000
C	5.389	-1.436	0.00	-0.040	-0.005	0.000	-0.044	0.014	-0.000
C	4.188	0.708	0.00	-0.077	0.127	-0.000	-0.026	0.053	0.000
C	4.188	-0.708	0.00	-0.044	-0.082	-0.000	-0.069	-0.111	0.000

H	7.549	1.243	0.00	0.059	-0.055	0.000	0.022	-0.020	-0.000
H	7.549	-1.243	0.00	0.035	0.032	0.000	0.052	0.048	-0.000
C	2.784	1.124	0.00	0.292	0.065	0.000	0.159	0.156	-0.000
C	2.784	-1.124	0.00	0.224	-0.167	0.000	0.245	-0.025	0.000
H	5.376	2.532	0.00	-0.011	-0.011	0.000	0.046	0.011	0.000
H	5.376	-2.532	0.00	0.042	-0.008	0.000	-0.022	0.014	0.000
N	2.395	-2.395	0.00	-0.099	0.087	-0.000	-0.038	-0.061	-0.000
C	0.708	-6.591	0.00	-0.013	-0.016	-0.000	0.015	0.035	0.000
N	-1.988	-0.000	0.00	-0.270	-0.014	0.000	-0.211	0.018	0.000
C	-0.708	-6.591	0.00	0.011	-0.030	0.000	-0.016	0.024	-0.000
N	-0.000	-1.988	0.00	0.018	0.211	0.000	0.014	-0.270	0.000
C	1.436	-5.389	0.00	0.008	0.028	0.000	0.012	-0.052	-0.000
C	-1.436	-5.389	0.00	0.014	0.044	-0.000	0.005	-0.040	0.000
C	0.708	-4.188	0.00	0.053	0.026	-0.000	-0.127	-0.077	0.000
C	-0.708	-4.188	0.00	-0.111	0.069	0.000	0.082	-0.044	0.000
H	1.243	-7.549	0.00	-0.020	-0.022	0.000	0.055	0.059	-0.000
H	-1.243	-7.549	0.00	0.048	-0.052	0.000	-0.032	0.035	-0.000
C	1.124	-2.784	0.00	0.156	-0.159	-0.000	-0.065	0.292	-0.000
C	-1.124	-2.784	0.00	-0.025	-0.245	-0.000	0.167	0.224	-0.000
H	2.532	-5.376	0.00	0.011	-0.046	-0.000	0.011	-0.011	0.000
C	-2.784	-1.124	0.00	0.292	0.065	0.000	0.159	0.156	-0.000
H	-2.532	-5.376	0.00	0.014	0.022	0.000	0.008	0.042	-0.000
C	-2.784	1.124	0.00	0.224	-0.167	-0.000	0.245	-0.025	-0.000
C	-4.188	-0.708	0.00	-0.077	0.127	0.000	-0.026	0.053	0.000
C	-4.188	0.708	0.00	-0.044	-0.082	-0.000	-0.069	-0.111	-0.000
C	-5.389	-1.436	0.00	-0.052	-0.012	-0.000	-0.028	0.008	-0.000
N	-2.395	-2.395	0.00	-0.061	0.038	-0.000	-0.087	-0.099	0.000

C	-5.389	1.436	0.00	-0.040	-0.005	0.000	-0.044	0.014	0.000
N	-2.395	2.395	0.00	-0.099	0.087	-0.000	-0.038	-0.061	0.000
C	-6.591	-0.708	0.00	0.035	-0.015	0.000	0.016	-0.013	0.000
C	-6.591	0.708	0.00	0.024	0.016	-0.000	0.030	0.011	-0.000
H	-5.376	-2.532	0.00	-0.011	-0.011	-0.000	0.046	0.011	0.000
H	-5.376	2.532	0.00	0.042	-0.008	-0.000	-0.022	0.014	-0.000
H	-7.549	-1.243	0.00	0.059	-0.055	-0.000	0.022	-0.020	-0.000
H	-7.549	1.243	0.00	0.035	0.032	0.000	0.052	0.048	-0.000
Cu	0.000	0.000	0.00	0.004	-0.003	0.000	0.003	0.004	-0.000

S7.4 1526 cm^{-1}

Table S6: Eigenvectors for the 1526 cm^{-1} eigenmode of CuPc. Each eigenvector is normalized to one and not mass-weighted. $\vec{a}_i$ and $\vec{b}_i$ correspond to the eigenvector components pointing into one of the two planar Cartesian coordinate directions. The positions of the atoms in space are denoted as x, y, z with units in Angstrom

Atom	x	y	z	$\vec{a}_x$	$\vec{a}_y$	$\vec{a}_z$	$\vec{b}_x$	$\vec{b}_y$	$\vec{b}_z$
C	0.708	4.188	0.00	-0.027	-0.024	-0.000	0.052	-0.024	-0.000
C	1.436	5.389	0.00	0.006	0.026	-0.000	-0.008	-0.017	0.000
C	0.708	6.591	0.00	0.004	-0.012	-0.000	-0.028	0.016	-0.000
C	-0.708	6.591	0.00	-0.004	0.008	0.000	0.028	0.018	-0.000
C	-1.436	5.389	0.00	0.003	-0.020	0.000	0.010	-0.023	0.000
C	-0.708	4.188	0.00	-0.012	0.029	-0.000	-0.057	-0.016	-0.000
H	1.243	7.549	0.00	-0.006	-0.008	-0.000	-0.006	0.002	0.000
H	-1.243	7.549	0.00	-0.007	0.007	-0.000	0.004	0.004	0.000
H	-2.532	5.376	0.00	0.004	0.056	-0.000	0.013	-0.024	-0.000
H	2.532	5.376	0.00	0.008	-0.047	0.000	-0.011	-0.039	0.000
C	-1.124	2.784	0.00	-0.038	-0.074	-0.000	0.387	0.100	0.000

N	0.000	1.988	0.00	-0.101	0.001	0.000	-0.015	-0.006	0.000
C	1.124	2.784	0.00	0.073	0.042	0.000	-0.382	0.117	-0.000
N	2.395	2.395	0.00	0.087	-0.211	-0.000	0.245	-0.153	-0.000
C	6.591	0.708	0.00	-0.018	0.028	-0.000	0.008	0.004	0.000
C	6.591	-0.708	0.00	-0.016	-0.028	-0.000	-0.012	-0.004	0.000
N	1.988	0.000	0.00	0.006	-0.015	-0.000	0.001	0.101	0.000
C	5.389	1.436	0.00	0.024	0.010	-0.000	-0.020	-0.003	-0.000
C	5.389	-1.436	0.00	0.017	-0.008	0.000	0.026	-0.006	-0.000
C	4.188	0.708	0.00	0.016	-0.057	-0.000	0.029	0.012	0.000
C	4.188	-0.708	0.00	0.024	0.052	-0.000	-0.024	0.027	0.000
H	7.549	1.243	0.00	-0.004	0.004	0.000	0.007	0.007	-0.000
H	7.549	-1.243	0.00	-0.002	-0.006	0.000	-0.008	0.006	-0.000
C	2.784	1.124	0.00	-0.100	0.387	0.000	-0.073	0.038	-0.000
C	2.784	-1.124	0.00	-0.117	-0.382	0.000	0.042	-0.073	0.000
H	5.376	2.532	0.00	0.024	0.013	0.000	0.056	-0.004	0.000
H	5.376	-2.532	0.00	0.039	-0.011	0.000	-0.047	-0.008	0.000
N	2.395	-2.395	0.00	0.153	0.245	-0.000	-0.211	-0.087	-0.000
C	0.708	-6.591	0.00	-0.004	0.008	-0.000	0.028	0.018	0.000
N	-1.988	-0.000	0.00	0.006	-0.015	0.000	0.001	0.101	0.000
C	-0.708	-6.591	0.00	0.004	-0.012	0.000	-0.028	0.016	-0.000
N	-0.000	-1.988	0.00	-0.101	0.001	0.000	-0.015	-0.006	0.000
C	1.436	-5.389	0.00	0.003	-0.020	0.000	0.010	-0.024	-0.000
C	-1.436	-5.389	0.00	0.006	0.026	-0.000	-0.008	-0.017	0.000
C	0.708	-4.188	0.00	-0.012	0.029	-0.000	-0.057	-0.016	0.000
C	-0.708	-4.188	0.00	-0.027	-0.024	0.000	0.052	-0.024	0.000
H	1.243	-7.549	0.00	-0.007	0.007	0.000	0.004	0.004	-0.000
H	-1.243	-7.549	0.00	-0.006	-0.008	0.000	-0.006	0.002	-0.000

C	1.124	-2.784	0.00	-0.038	-0.074	-0.000	0.387	0.100	-0.000
C	-1.124	-2.784	0.00	0.073	0.042	-0.000	-0.381	0.117	-0.000
H	2.532	-5.376	0.00	0.004	0.056	-0.000	0.013	-0.024	0.000
C	-2.784	-1.124	0.00	-0.100	0.387	0.000	-0.074	0.038	-0.000
H	-2.532	-5.376	0.00	0.008	-0.047	0.000	-0.011	-0.039	-0.000
C	-2.784	1.124	0.00	-0.117	-0.382	-0.000	0.042	-0.073	-0.000
C	-4.188	-0.708	0.00	0.016	-0.057	0.000	0.030	0.012	0.000
C	-4.188	0.708	0.00	0.024	0.052	-0.000	-0.024	0.027	-0.000
C	-5.389	-1.436	0.00	0.024	0.010	-0.000	-0.020	-0.003	-0.000
N	-2.395	-2.395	0.00	0.087	-0.211	-0.000	0.245	-0.153	0.000
C	-5.389	1.436	0.00	0.017	-0.008	0.000	0.026	-0.006	0.000
N	-2.395	2.395	0.00	0.153	0.245	-0.000	-0.211	-0.087	0.000
C	-6.591	-0.708	0.00	-0.018	0.028	0.000	0.008	0.004	0.000
C	-6.591	0.708	0.00	-0.016	-0.028	-0.000	-0.012	-0.004	-0.000
H	-5.376	-2.532	0.00	0.024	0.013	-0.000	0.056	-0.004	0.000
H	-5.376	2.532	0.00	0.039	-0.011	-0.000	-0.047	-0.008	-0.000
H	-7.549	-1.243	0.00	-0.004	0.004	-0.000	0.007	0.007	-0.000
H	-7.549	1.243	0.00	-0.002	-0.006	0.000	-0.008	0.006	-0.000
Cu	0.000	0.000	0.00	-0.003	-0.001	0.000	-0.001	0.003	-0.000

S7.5 3144 cm^{-1}

Table S7: Eigenvectors for the 3144 cm^{-1} eigenmode of CuPc. Each eigenvector is normalized to one and not mass-weighted. $\vec{a}_i$ and $\vec{b}_i$ correspond to the eigenvector components pointing into one of the two planar Cartesian coordinate directions. The positions of the atoms in space are denoted as x, y, z with units in Angstrom

**Atom**	**x**	**y**	**z**	$\vec{a}_x$	$\vec{a}_y$	$\vec{a}_z$	$\vec{b}_x$	$\vec{b}_y$	$\vec{b}_z$
C	0.708	4.188	0.00	-0.000	-0.002	-0.000	-0.000	-0.000	-0.000

C	1.436	5.389	0.00	0.039	-0.001	-0.000	0.011	-0.000	0.000
C	0.708	6.591	0.00	0.009	0.018	-0.000	0.002	0.005	-0.000
C	-0.708	6.591	0.00	-0.009	0.018	0.000	-0.002	0.004	-0.000
C	-1.436	5.389	0.00	-0.040	-0.001	0.000	-0.008	-0.000	0.000
C	-0.708	4.188	0.00	0.000	-0.002	-0.000	0.000	-0.000	-0.000
H	1.243	7.549	0.00	-0.105	-0.189	-0.000	-0.027	-0.049	0.000
H	-1.243	7.549	0.00	0.106	-0.191	-0.000	0.023	-0.041	0.000
H	-2.532	5.376	0.00	0.449	0.003	-0.000	0.085	0.001	-0.000
H	2.532	5.376	0.00	-0.440	0.003	0.000	-0.125	0.001	0.000
C	-1.124	2.784	0.00	-0.000	-0.000	-0.000	-0.000	-0.000	0.000
N	0.000	1.988	0.00	-0.000	0.000	0.000	0.000	0.000	0.000
C	1.124	2.784	0.00	0.000	-0.000	0.000	0.000	-0.000	-0.000
N	2.395	2.395	0.00	-0.000	-0.000	-0.000	-0.000	0.000	-0.000
C	6.591	0.708	0.00	0.004	0.002	-0.000	-0.018	-0.009	0.000
C	6.591	-0.708	0.00	0.005	-0.002	-0.000	-0.018	0.009	0.000
N	1.988	0.000	0.00	0.000	-0.000	-0.000	-0.000	-0.000	0.000
C	5.389	1.436	0.00	-0.000	0.008	-0.000	0.001	-0.039	-0.000
C	5.389	-1.436	0.00	-0.000	-0.011	0.000	0.001	0.039	-0.000
C	4.188	0.708	0.00	-0.000	-0.000	-0.000	0.002	0.000	0.000
C	4.188	-0.708	0.00	-0.000	0.000	-0.000	0.002	-0.000	0.000
H	7.549	1.243	0.00	-0.041	-0.023	0.000	0.187	0.104	-0.000
H	7.549	-1.243	0.00	-0.049	0.027	0.000	0.185	-0.103	-0.000
C	2.784	1.124	0.00	-0.000	0.000	0.000	0.000	-0.000	-0.000
C	2.784	-1.124	0.00	-0.000	-0.000	0.000	0.000	0.000	0.000
H	5.376	2.532	0.00	0.001	-0.085	0.000	-0.003	0.439	0.000
H	5.376	-2.532	0.00	0.001	0.125	0.000	-0.003	-0.430	0.000
N	2.395	-2.395	0.00	0.000	0.000	-0.000	0.000	-0.000	-0.000

C	0.708	-6.591	0.00	-0.009	0.018	-0.000	-0.002	0.004	0.000
N	-1.988	-0.000	0.00	0.000	-0.000	0.000	-0.000	-0.000	0.000
C	-0.708	-6.591	0.00	0.009	0.017	0.000	0.002	0.005	-0.000
N	-0.000	-1.988	0.00	-0.000	0.000	0.000	0.000	0.000	0.000
C	1.436	-5.389	0.00	-0.039	-0.001	0.000	-0.007	-0.000	-0.000
C	-1.436	-5.389	0.00	0.038	-0.001	-0.000	0.011	-0.000	0.000
C	0.708	-4.188	0.00	0.000	-0.002	-0.000	0.000	-0.000	0.000
C	-0.708	-4.188	0.00	-0.000	-0.002	0.000	-0.000	-0.000	0.000
H	1.243	-7.549	0.00	0.102	-0.183	0.000	0.022	-0.039	-0.000
H	-1.243	-7.549	0.00	-0.101	-0.181	0.000	-0.026	-0.047	-0.000
C	1.124	-2.784	0.00	-0.000	-0.000	-0.000	-0.000	-0.000	-0.000
C	-1.124	-2.784	0.00	0.000	-0.000	-0.000	0.000	-0.000	-0.000
H	2.532	-5.376	0.00	0.429	0.003	-0.000	0.080	0.001	0.000
C	-2.784	-1.124	0.00	-0.000	0.000	0.000	0.000	-0.000	-0.000
H	-2.532	-5.376	0.00	-0.420	0.003	0.000	-0.121	0.001	-0.000
C	-2.784	1.124	0.00	-0.000	-0.000	-0.000	0.000	0.000	-0.000
C	-4.188	-0.708	0.00	-0.000	-0.000	0.000	0.002	0.000	0.000
C	-4.188	0.708	0.00	-0.000	0.000	-0.000	0.002	-0.000	-0.000
C	-5.389	-1.436	0.00	-0.000	0.007	-0.000	0.001	-0.039	-0.000
N	-2.395	-2.395	0.00	-0.000	-0.000	-0.000	-0.000	0.000	0.000
C	-5.389	1.436	0.00	-0.000	-0.011	0.000	0.001	0.039	0.000
N	-2.395	2.395	0.00	0.000	0.000	-0.000	0.000	-0.000	0.000
C	-6.591	-0.708	0.00	0.004	0.002	0.000	-0.018	-0.009	0.000
C	-6.591	0.708	0.00	0.005	-0.002	-0.000	-0.018	0.009	-0.000
H	-5.376	-2.532	0.00	0.001	-0.080	-0.000	-0.003	0.440	0.000
H	-5.376	2.532	0.00	0.001	0.121	-0.000	-0.003	-0.430	-0.000
H	-7.549	-1.243	0.00	-0.039	-0.022	-0.000	0.187	0.104	-0.000

H	-7.549	1.243	0.00	-0.047	0.026	0.000	0.185	-0.103	-0.000
Cu	0.000	0.000	0.00	-0.000	-0.000	0.000	0.000	-0.000	-0.000

References

(1) Bannwarth, C.; Ehlert, S.; Grimme, S. GFN2-xTB—An Accurate and Broadly Parametrized Self-Consistent Tight-Binding Quantum Chemical Method with Multipole Electrostatics and Density-Dependent Dispersion Contributions. *Journal of Chemical Theory and Computation* **2019**, *15*, 1652–1671.

(2) Bannwarth, C.; Caldeweyher, E.; Ehlert, S.; Hansen, A.; Pracht, P.; Seibert, J.; Spicher, S.; Grimme, S. Extended tight-binding quantum chemistry methods. *WIREs Computational Molecular Science* **2021**, *11*, e1493.

(3) Epifanovsky, E.; et al. Software for the frontiers of quantum chemistry: An overview of developments in the Q-Chem 5 package. *The Journal of Chemical Physics* **2021**, *155*, 084801.

(4) Darling, B. T.; Dennison, D. M. The Water Vapor Molecule. *Phys. Rev.* **1940**, *57*, 128–139.

(5) Watson, J. K. Simplification of the molecular vibration-rotation hamiltonian. *Molecular Physics* **1968**, *15*, 479–490.

(6) Moss, R.; Perry, A. The vibrational Zeeman effect. *Molecular Physics* **1973**, *25*, 1121–1134.

(7) Gauss, J.; Ruud, K.; Helgaker, T. Perturbation-dependent atomic orbitals for the calculation of spin-rotation constants and rotational g tensors. *The Journal of Chemical Physics* **1996**, *105*, 2804–2812.

(8) Wang, B.; Tam, C. N.; Keiderling, T. A. Vibrational Zeeman effect for the ν_4 mode of haloforms (HCX_3) determined by magnetic vibrational circular dichroism. *Phys. Rev. Lett.* **1993**, *71*, 979–982.

(9) Wang, B.; Keiderling, T. A. Measurement of the vibrational Zeeman effect for HCF3 using magnetic vibrational circular dichroism. *The Journal of Chemical Physics* **1994**, *101*, 905–911.

(10) Dauxois, T.; Peyrard, M. *Physics of Solitons*; Cambridge University Press, 2006.
